# WaterMamba: A Hybrid Mamba-Enhanced Detection Transformer for Small River Obstacle Detection in Complex Hydrological Environments

**DOI:** 10.3390/s26196187

**Published:** 2026-09-29

**Authors:** Xiang Li, Lang Qin, Li Ren

**Affiliations:** College of Hydrology and Water Resources, Hohai University, Nanjing 210098, China; 2416010119@hhu.edu.cn (X.L.); 2324010115@hhu.edu.cn (L.Q.)

**Keywords:** river obstacle detection, state space model, Mamba, small target detection, frequency-domain attention, water scene understanding

## Abstract

Small river obstacles are difficult to distinguish from reflections, ripples, turbidity, and low-contrast backgrounds, while spatial-only detectors may inadequately preserve small-target detail and long-range water-surface context. We propose WaterMamba, a hybrid Mamba-enhanced detection transformer built on RT-DETR. The model combines a MobileMamba backbone with Multi-Receptive Field Feature Interaction (MRFFI), a Hybrid Spatial-Spectral Mamba (HybridMamba) encoder with Frequency-Domain Attention (FDA), a Triple-Channel Window Block (TCWB) for multi-scale fusion, and an Adaptive Scale-Aware Detection Loss (ASDL). Under a unified RGB-only protocol, WaterMamba improves over RT-DETR-L by 2.8 percentage points in mAP_50_ and 4.5 points in AP_*S*_ on WaterScenes; the corresponding AP_*S*_ gains on LaRS and IWHR are 4.8 points. Full-evaluation-set COCO-style metrics and controlled ablations establish the quantitative comparison. At the common confidence threshold of 0.3, the qualitative panels complement these statistics by showing the operating-point behavior on difficult small and low-contrast instances; similarity among boxes jointly detected by strong models is expected and does not negate differences in difficult-target recall. WaterMamba contains 28.6 M parameters, requires 96.2 G FLOPs, and reaches 78.4 FPS on an NVIDIA A100; embedded-platform latency remains outside the present evaluation. The source code is available at the repository specified in the Data Availability Statement.

## 1. Introduction

Inland waterway transportation constitutes a vital component of global logistics, offering an economical and environmentally sustainable transport mode. With the advancement of autonomous surface vehicles (ASVs) and intelligent water management systems, ensuring safe navigation through automatic river obstacle detection has become a critical research priority [1]. River obstacles, including floating debris, small watercraft, buoys, swimmers, and partially submerged hazards, pose significant threats to navigation safety in complex hydrological scenarios with turbid water, variable illumination, and dynamic conditions.

China possesses over 127,000 km of navigable inland waterways, the largest network in the world, carrying approximately 3.7 billion tonnes of freight annually. In recent years, the Chinese government has prioritized intelligent waterway infrastructure, promoting the deployment of unmanned patrol vessels and automated monitoring stations for real-time hazard awareness. These systems rely critically on robust visual detection algorithms capable of operating under the full spectrum of hydrological conditions encountered across diverse river basins, ranging from the sediment-laden Yellow River to the monsoon-affected Yangtze tributaries. The heterogeneity of these environments, combined with the prevalence of small and irregularly shaped obstacles, creates a pressing need for detection frameworks that go beyond general-purpose architectures.

Detecting small-scale river obstacles presents unique challenges that fundamentally differ from general object detection. First, obstacles such as distant buoys, floating branches, and partially submerged objects occupy extremely small pixel regions (often below 32×32 pixels), making them easily confused with background clutter [2,3]. According to COCO-style evaluation, small objects account for over 60% of all obstacle instances in typical waterway monitoring imagery, yet contribute disproportionately less to overall detection metrics due to their inherent difficulty. Second, the complex water surface introduces substantial visual interference through specular reflections, wave-induced deformations, and varying turbidity, creating spurious feature responses that frequently trigger false positives. Third, significant illumination variations between day and night, combined with weather-dependent factors such as fog, rain, and surface glare, further compound the difficulty. Fourth, the aspect ratios and morphologies of river obstacles exhibit high variability, ranging from elongated floating logs to near-circular buoys, demanding flexible feature representations.

Recent advances in object detection, from Faster R-CNN [4] to the YOLO series [5,6,7,8,9,10], have achieved impressive performance. Detection Transformers (DETR) [11,12,13] introduced end-to-end set prediction, and RT-DETR [14] bridged the efficiency gap by combining CNN backbones with hybrid encoders. Recent scene-specific benchmarks also emphasize that detection performance should be assessed under geographic, weather, viewpoint, and clutter variations rather than only on generic data. For example, RSUD20K [15] evaluates contemporary detectors on high-resolution, geographically diverse road scenes containing dense objects and adverse conditions. Although road and water-surface imagery differ substantially, this benchmark highlights the importance of analyzing domain-specific background interference and scale variation. Nevertheless, small-target detection in water scenes remains difficult because targets contain limited appearance information and are easily confused with dynamic backgrounds [2].

State Space Models (SSMs), particularly Mamba [16], have emerged as a compelling alternative to Transformers. Built upon structured state spaces [17], Mamba introduces selective scanning whose sequence-processing cost scales linearly with sequence length under its stated implementation assumptions. Visual adaptations including VMamba [18] and Vision Mamba [19] have demonstrated strong potential in image understanding. Their content-dependent long-range modeling motivates our investigation of selective state-space processing for water-scene detection.

We propose WaterMamba, a hybrid Mamba-enhanced detection transformer for small-target obstacle detection in complex hydrological scenarios. Our design tests a bounded hypothesis: spatial representations can be complemented by learned frequency-domain processing because water-surface interference and obstacle boundaries distribute energy differently across spatial frequencies. The proposed FDA does not impose a hand-crafted frequency cutoff; instead, it learns channel-dependent spectral modulation within the detector. We evaluate whether this design improves full-set detection metrics through controlled comparisons and ablations rather than inferring performance from selected images.

WaterMamba employs a MobileMamba backbone with MRFFI for efficient multi-scale extraction using bidirectional Mamba and multi-range convolutions. The encoder features HybridMamba, which integrates VSSM, MRFB, Multi-Branch Hybrid Attention (MBHA), and an FDA branch adapted from prior spectral–spatial detector design [20] for single-modal RGB water scenes. TCWB handles multi-scale fusion through three complementary attention channels. An Adaptive Scale-Aware Detection Loss (ASDL) assigns higher weights to small targets during optimization.

Our main contributions and the provenance of the reused backbone component are summarized as follows:

- We propose WaterMamba, a hybrid Mamba-enhanced detection transformer integrating state space models with frequency-domain analysis for small-target river obstacle detection.
- We adopt the MobileMamba backbone and its MRFFI module from prior work [21] and integrate this established feature extractor into the RT-DETR framework; MRFFI is not claimed as an original contribution of WaterMamba.
- We develop the HybridMamba configuration for single-modal RGB water scenes by combining VSSM, MRFB, MBHA, and an FDA branch adapted from prior spectral–spatial detection work [20]; the contribution is this task-specific configuration and evaluation rather than the original invention of FDA.
- We propose TCWB for enhanced multi-scale feature fusion via parallel triple-channel window attention.
- We design ASDL with scale-dependent weighting for improved small-target sensitivity.
- We evaluate the detector through full-set COCO-style metrics, controlled component ablations, cross-dataset comparisons, and fixed-threshold qualitative examples of difficult small or low-contrast targets.

## 2. Related Work

### 2.1. General Object Detection

Modern object detection follows two paradigms. The two-stage paradigm, including Faster R-CNN [4], Cascade R-CNN [22], Dynamic R-CNN [23], and Sparse R-CNN [24], achieves high accuracy at greater computational cost. The one-stage paradigm directly predicts detections, with anchor-based methods such as RetinaNet [25] and YOLO variants [6,8,10], and anchor-free approaches such as FCOS [26]. Feature Pyramid Networks [27] have become standard for multi-scale representation.

DETR [11] introduced transformer-based end-to-end detection. Subsequent works improved upon it: Deformable DETR [12] introduced efficient multi-scale attention; DAB-DETR [28] and DN-DETR [29] improved query formulation; DINO [13] integrated contrastive denoising; CO-DETR [30] employed collaborative hybrid assignments. RT-DETR [14] achieved real-time DETR performance by designing an efficient hybrid encoder that decouples intra-scale interaction and cross-scale fusion, and introducing IoU-aware query selection. RSUD20K [15] further demonstrates the value of evaluating modern detectors in domain-specific scenes with high resolution, crowded objects, and geographic variation. RT-DETR serves as our baseline due to its favorable balance between accuracy and efficiency, and its end-to-end nature that eliminates the need for NMS post-processing.

### 2.2. Small Object Detection

Small object detection remains a long-standing challenge in computer vision. Early approaches focused on multi-scale feature aggregation through Feature Pyramid Networks [27] and their variants. Data augmentation strategies such as copy-paste augmentation [3] and mosaic training have proven effective for alleviating the scarcity of small object training samples. More recently, attention-based mechanisms have been explored to enhance small object feature representation. Cheng et al. [2] conducted a comprehensive survey on small object detection in unmanned surface vehicle navigation, identifying key challenges including limited appearance information, background confusion, and scale imbalance. Loss function design also plays a critical role: scale-aware reweighting strategies [25] and IoU-based loss functions [31] have been proposed to address the inherent imbalance between small and large objects during training. Despite these advances, most small object detection methods operate in the spatial domain alone, overlooking the potential of frequency-domain analysis for distinguishing small targets from background interference. Our ASDL loss and water-scene adaptation of FDA address this limitation.

### 2.3. State Space Models for Vision

Structured State Space Models (S4) [17] established efficient long-range sequence modeling via structured matrix parameterization. Mamba [16] introduced selective scanning with input-dependent parameters and linear sequence-length scaling. Mamba-2 [32] unified SSMs with attention through structured duality. In vision, VMamba [18] pioneered cross-scan adaptation; Vision Mamba [19] proposed bidirectional encoding; and MobileMamba [21] introduced a lightweight multi-receptive architecture, including the MRFFI module adopted in our backbone.

### 2.4. Water Scene Object Detection

Water scene detection has gained momentum with autonomous maritime systems. WaterScenes [33] provides a comprehensive multi-task water-scene benchmark with diverse categories and conditions; LaRS [34] offers panoptic maritime obstacle detection across harbors, open seas, and coastal areas; and USVInland [1] specifically targets unmanned surface vehicles in inland waterways. These domain-relevant datasets and studies establish the small-object, background-confusion, and environmental-variability challenges addressed in this work.

Despite these advances, most detector comparisons in water scenes remain dominated by spatial architectures transferred from general-image benchmarks. Water surfaces contain scene-dependent mixtures of slowly varying reflections, repetitive ripples, glare, and attenuated texture. These patterns motivate frequency-domain processing as a complementary representation, but they do not imply that a fixed band is universally separable from obstacle content. WaterMamba therefore couples learned spectral modulation with spatial state-space modeling and evaluates the resulting design through full-set comparisons and component ablations. This positioning distinguishes the proposed method from spatial-only detectors while keeping the physical interpretation within the evidence provided by the present experiments.

## 3. Proposed Method

### 3.1. Overall Architecture

**Figure 1:**
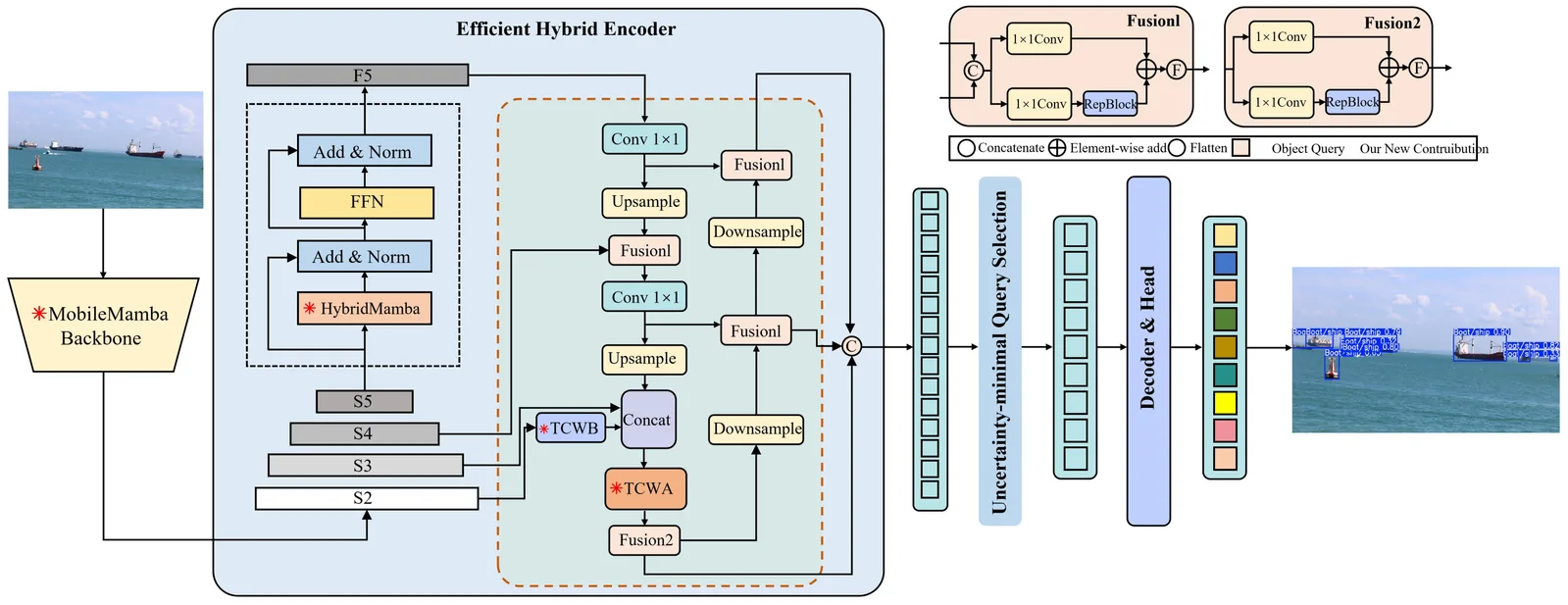
Overall architecture of WaterMamba. The framework comprises a MobileMamba backbone, an Efficient Hybrid Encoder with HybridMamba and TCWB modules (marked with *), top-scoring encoder-feature query selection, and Decoder and Head. The public implementation uses 1×1 projection, top-down feature addition, and TCWB-based refinement for cross-scale aggregation. Circled C denotes concatenation, whereas circled F denotes flattening.

The architecture of WaterMamba is shown in Figure 1. Built upon RT-DETR, it comprises four principal components: (1) a MobileMamba backbone for multi-scale feature extraction at four pyramid levels; (2) an Efficient Hybrid Encoder with HybridMamba and TCWB for intra-scale modeling and cross-scale fusion; (3) RT-DETR-style top-scoring encoder-feature selection for informative query initialization; and (4) a transformer decoder with a detection head for final box and class prediction.

The overall design philosophy is to address the specific challenges of river obstacle detection through targeted, task-aware components. The MobileMamba backbone is chosen because its selective state-space scans can model long-range spatial dependencies across large feature maps without global self-attention at those stages. HybridMamba is positioned at the top encoder level where feature resolution is lowest but semantic richness is highest, making it the most appropriate stage for frequency-domain spectral analysis. TCWB is applied during cross-scale refinement, including the lower feature levels where fine-grained spatial detail is still available, and its triple-channel design enables simultaneous multi-scale structural and positional reasoning. Finally, the ASDL loss operates on the training objective rather than the architecture, ensuring that scale awareness is enforced throughout the entire optimization process rather than at a single architectural bottleneck.

The linear sequence-length scaling stated for selective Mamba scanning applies only to that operation. WaterMamba as a whole also contains FFT/IFFT, multi-head window attention, multi-kernel convolutions, feature-pyramid fusion, and a transformer decoder. We therefore make no claim that the complete detector has globally linear complexity in image size; its practical cost is instead characterized by the reported model-level FLOPs and measured throughput under the specified input resolution and hardware.

To clarify the relation between WaterMamba and existing architectures, we regard RT-DETR as the detection framework on which the query selection, decoder, and prediction head are retained. The MobileMamba backbone and its MRFFI module are adopted from the original MobileMamba work [21]; MobileMamba-DETR provides prior precedent for integrating an MRFFI-enhanced MobileMamba backbone and frequency-domain attention into a DETR-style detector [20]. We therefore do not claim MRFFI or the general FDA concept as original modules. The task-specific contributions of WaterMamba are the HybridMamba configuration and FDA adaptation for single-modal RGB water scenes, the TCWB fusion block, ASDL, and their combined evaluation under a unified water-scene protocol.

Given input $I\in R^{H\times W\times 3}$, the backbone extracts $\left\{S_{2},S_{3},S_{4},S_{5}\right\}$ at strides {4,8,16,32}. The top-level feature $F_{5}$ is processed through HybridMamba. Features are fused across scales via the Efficient Hybrid Encoder, where each feature level is first projected to the hidden dimension and lower-level features are refined by adding the nearest-neighbor upsampled higher-level feature followed by TCWB. Top-scoring encoder features are selected as object queries for the decoder, which produces final detections.

### 3.2. MobileMamba Backbone with MRFFI

**Figure 2:**
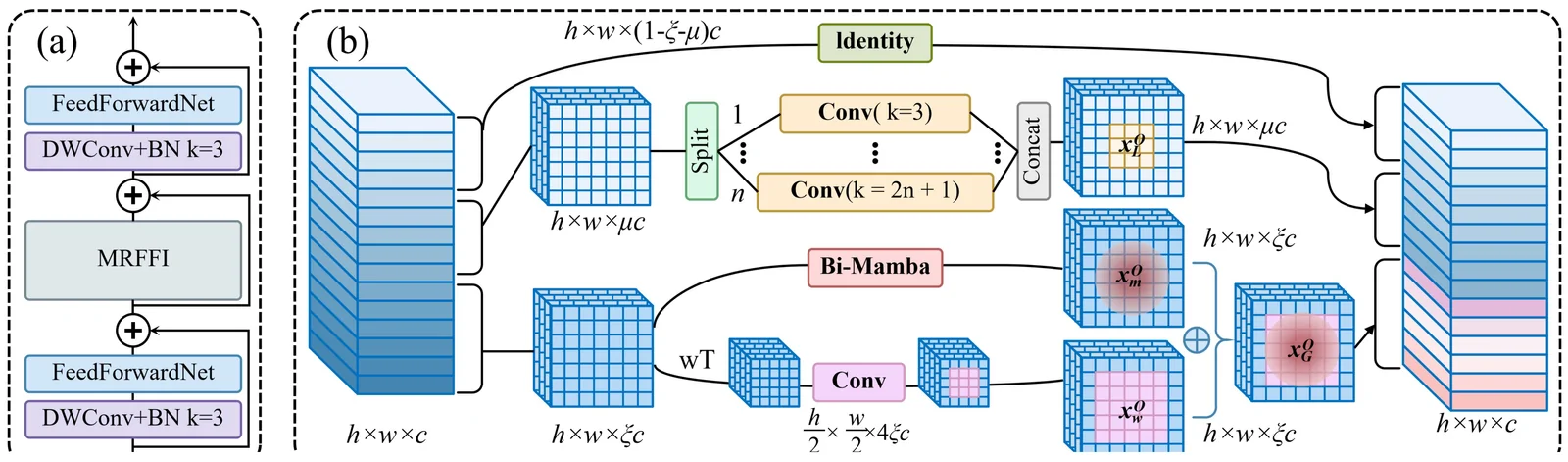
(**a**) MobileMamba block with alternating FeedForwardNet and MRFFI modules. (**b**) Multi-Receptive Field Feature Interaction (MRFFI) module adopted from MobileMamba [21]; its use in a DETR-style detector is also described in MobileMamba-DETR [20].

Each MobileMamba block (Figure 2a) consists of MRFFI-based feature interaction, normalization, and feed-forward convolutional refinement with residual connections.

MobileMamba was selected as the backbone for three practical reasons. First, its selective state-space operation provides long-range feature mixing with linear sequence-length scaling at the scan operation, which is useful for high-resolution water-scene feature maps relative to global self-attention in the early backbone stages. Second, MRFFI combines depthwise convolutions at multiple receptive-field sizes with bidirectional scanning, allowing the backbone to represent both compact obstacle edges and broader water-surface context. Third, compared with replacing RT-DETR-L with a heavier transformer backbone, MobileMamba reduces parameters and FLOPs while retaining multi-scale feature outputs compatible with the RT-DETR encoder. This makes it an appropriate backbone for a single-modal RGB setting where the detector must use subtle texture, shape, and context cues without relying on infrared or depth information.

The MRFFI formulation is adopted from MobileMamba [21] and is used here as an established backbone component rather than a new WaterMamba module. In the released implementation, MRFFI (Figure 2b) operates through parallel local, medium-range, large-range, and Haar-style rearrangement pathways on input $x\in R^{c\times h\times w}$:

**Multi-Range Convolution Path.** The input is processed by depthwise convolutions with kernel sizes 3, 5, and 7:

(1) $$x_{\mathrm{conv}}=\left[{\mathrm{DWConv}}_{3}\left(x\right);{\mathrm{DWConv}}_{5}\left(x\right);{\mathrm{DWConv}}_{7}\left(x\right)\right].$$

The three branches provide local structural responses at different receptive-field sizes.

**Haar-Style Rearrangement and Bidirectional Scan.** In our implementation, the wavelet-inspired transform (wT) is a fixed Haar-style spatial rearrangement rather than a learned wavelet layer. For each channel, four sub-bands are formed from adjacent 2×2 locations:

(2) $$\begin{array}{cc} z_{LL} & =(x_{00}+x_{01}+x_{10}+x_{11})/2,\\ z_{LH} & =(x_{00}-x_{01}+x_{10}-x_{11})/2,\\ z_{HL} & =(x_{00}+x_{01}-x_{10}-x_{11})/2,\\ z_{HH} & =(x_{00}-x_{01}-x_{10}+x_{11})/2, \end{array}$$

where $x_{00},x_{01},x_{10},x_{11}$ denote the even-even, even-odd, odd-even, and odd-odd spatial samples. The four sub-bands are concatenated along the channel dimension, resized back to the original feature resolution, and concatenated with the three depthwise-convolution branches. A 1×1 convolution compresses the combined tensor before the bidirectional selective scan:

(3) $$x_{\mathrm{scan}}=\mathrm{BiScan}\;\left({\mathrm{Conv}}_{1\times 1}\left(\left[x_{\mathrm{conv}};\mathrm{Resize}\left(\mathrm{wT}\left(x\right)\right)\right]\right)\right).$$

The MRFFI output is produced by a residual update followed by feed-forward convolutional refinement:

(4) $$x_{\mathrm{out}}=y+\mathrm{FFN}\left(y\right),\;y=x+\mathrm{BN}\left(x_{\mathrm{scan}}\right).$$

This implementation keeps the transform deterministic and reproducible while retaining the multi-range and bidirectional sequence-mixing behavior of the adopted MRFFI design.

### 3.3. Hybrid Spatial-Spectral Mamba

**Figure 3:**
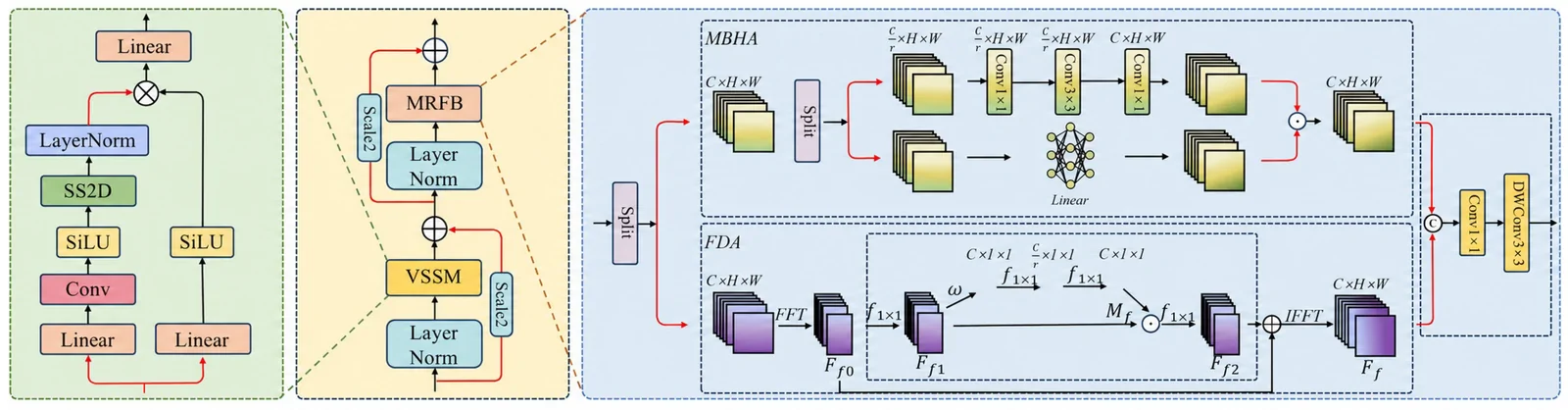
Hybrid Spatial-Spectral Mamba architecture. (**Left**): VSSM with SS2D scanning. (**Middle**): Overall HybridMamba combining VSSM and MRFB through residual feature mixing. (**Right-Top**): MBHA module. (**Right-Bottom**): FDA module.

HybridMamba (Figure 3) replaces standard encoder attention with a hybrid architecture that jointly models spatial structures through VSSM and spectral characteristics through FDA, connected by MRFB and MBHA. The FDA branch adapts the frequency-domain attention concept used in MobileMamba-DETR [20]; its integration with VSSM, MRFB, and MBHA is configured here for single-modal RGB water scenes.

The configuration is intended to avoid relying on a single representation pathway. VSSM provides efficient long-range spatial dependency modeling; MRFB restores local and medium-range geometric detail that may be weakened by sequence-style scanning; MBHA supplies branch-wise spatial attention over different kernel ranges; and FDA provides a learnable spectral modulation path for water-surface patterns such as glare, ripples, and turbidity. For RGB water scenes, these cues are complementary: small obstacles require local boundary detail, while the surrounding water background often requires broader contextual and spectral discrimination. HybridMamba therefore integrates these components at the semantic encoder level, where the feature map is compact enough for spectral processing but still retains task-relevant spatial structure.

#### 3.3.1. Visual State Space Model (VSSM)

The VSSM builds upon the continuous-time state space formulation. A linear time-invariant system maps input u(t) to output y(t) through a hidden state h(t):

(5) $$h^{\prime }\left(t\right)=Ah\left(t\right)+Bu\left(t\right),\;y\left(t\right)=Ch\left(t\right)$$

where $A\in R^{N\times N}$, $B\in R^{N\times 1}$, $C\in R^{1\times N}$ are learnable parameters. For discrete processing, the system is discretized using a step size Δ:

(6) $$h_{k}=\bar{A}h_{k-1}+\bar{B}u_{k},\;y_{k}=Ch_{k}$$

where $\bar{A}=\exp\left(\Delta A\right)$ and $\bar{B}={\left(\Delta A\right)}^{-1}\left(\exp\left(\Delta A\right)-I\right)\cdot \Delta B$. The selective mechanism makes B, C, and Δ input-dependent via learned projections:

(7) $$B_{k}=s_{B}\left(x_{k}\right),\;C_{k}=s_{C}\left(x_{k}\right),\;\Delta_{k}=\mathrm{softplus}\left(s_{\Delta }\left(x_{k}\right)\right)$$

For 2D image features, the SS2D module scans along four directional paths (left-to-right, right-to-left, top-to-bottom, bottom-to-top) and merges the results. In the released implementation, feature maps are flattened into tokens, normalized by LayerNorm, processed by four directional selective scans, fused by a linear projection, and modulated by an input-dependent sigmoid gate before the residual update.

#### 3.3.2. Multi-Range Fusion Block (MRFB)

The MRFB provides multi-range feature enhancement that complements the VSSM. Given input x, the released implementation applies depthwise convolutions with kernel sizes 3, 5, and 7, concatenates their responses, and fuses them using a 1×1 projection followed by batch normalization and GELU:

(8) $$\mathrm{MRFB}\left(x\right)=\phi \;\left(\mathrm{BN}\;\left({\mathrm{Conv}}_{1\times 1}\left([{\mathrm{DWConv}}_{3}\left(x\right);{\mathrm{DWConv}}_{5}\left(x\right);{\mathrm{DWConv}}_{7}\left(x\right)]\right)\right)\right),$$

where ϕ denotes GELU. This merge strategy captures patterns at multiple spatial ranges simultaneously, enriching the VSSM output with local structural details that complement its global modeling capability.

#### 3.3.3. Multi-Branch Hybrid Attention (MBHA)

MBHA (Figure 3, right-top) captures multi-scale spatial interactions through parallel branches. The input $x\in R^{C\times H\times W}$ is split along the channel dimension into four groups. The groups are processed by convolutions with kernel sizes 1×1, 3×3, 5×5, and 7×7, followed by batch normalization and GELU. Their concatenated response is combined with a residual depthwise-convolution branch and modulated by a learned sigmoid attention map:

(9) $$x_{\mathrm{mbha}}=x+{\mathrm{Conv}}_{1\times 1}\;\left(M⊙\sigma ({\mathrm{Conv}}_{1\times 1}\left(M\right))\right),\;M={\left[{\mathrm{Conv}}_{k_{s}}\left(x^{\left(s\right)}\right)\right]}_{s=1}^{4}+{\mathrm{DWConv}}_{3\times 3}\left(x\right).$$

where σ is sigmoid activation and ⊙ denotes element-wise multiplication. The residual DWConv branch preserves spatial details.

#### 3.3.4. Frequency-Domain Attention (FDA)

FDA (Figure 3, right-bottom) is used as a task-adapted spectral branch within HybridMamba, rather than as a newly proposed general attention mechanism. Water-surface imagery contains mixtures of reflections, ripples, turbidity, and object boundaries whose useful cues are not always separable by a fixed frequency band. FDA therefore operates in the frequency domain to learn channel-dependent spectral modulation jointly with the spatial branches.

Given input $F\in R^{C\times H\times W}$, the FDA first applies a 2D Fast Fourier Transform independently to each channel:

(10) $$F_{f_{0}}={\mathrm{FFT}}_{2D}\left(F\right)$$

The complex-valued spectrum is represented by concatenating its real and imaginary parts along the channel dimension, producing a real tensor in $R^{2C\times H\times W}$. The frequency representation is then processed through learnable 1×1 convolutions that generate a channel-dependent spectral mask:

(11) $$M_{F}=\sigma \;\left({\mathrm{Conv}}_{1\times 1}^{\left(2\right)}\;\left(\phi \;\left({\mathrm{Conv}}_{1\times 1}^{\left(1\right)}\left(\left[ℜ\left(F_{f_{0}}\right);ℑ\left(F_{f_{0}}\right)\right]\right)\right)\right)\right).$$

The filtered frequency features are transformed to the spatial domain via inverse FFT:

(12) $$F_{f}=ℜ\left[{\mathrm{IFFT}}_{2D}\;\left(M_{F}⊙F_{f_{0}}\right)\right].$$

The FDA output is projected by a 1×1 convolution, normalized by batch normalization, and added back through a residual connection, so the module can fall back to spatial features when spectral cues are weak. The FFT and IFFT use orthonormal normalization, and no hand-crafted frequency threshold is imposed. This design enables FDA to learn frequency-selective modulation that can reduce water-specific spectral interference while preserving obstacle-discriminative frequency components. The learnable 1×1 convolutions in the frequency domain effectively act as channel-wise spectral filters, allowing each feature channel to learn its own frequency response profile.

### 3.4. Triple-Channel Window Block (TCWB)

**Figure 4:**
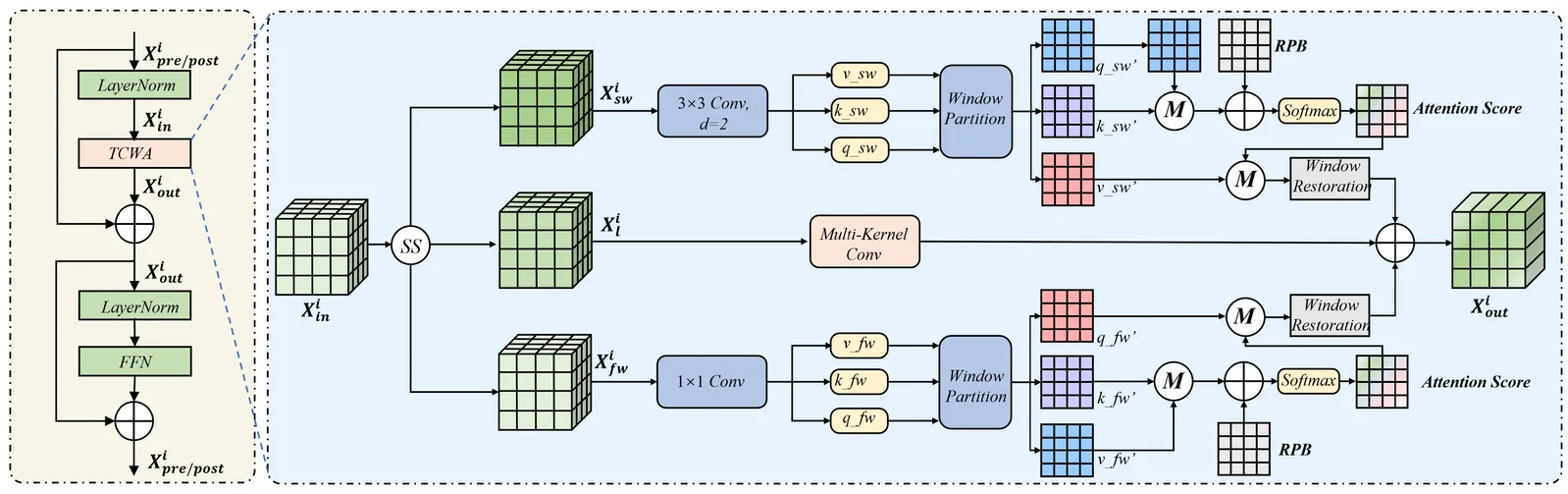
Triple-Channel Window Block (TCWB) structure. The input is split into three channels via Channel Soft Split (SS): a standard-window branch ($X_{sw}^{l}$), a multi-kernel branch ($X_{l}^{l}$), and a frequency-aware window branch ($X_{fw}^{l}$). Each branch applies distinct attention mechanisms before Window Restoration and concatenation. M denotes matrix multiplication, the circled plus sign denotes element-wise addition, and RPB denotes relative position bias.

TCWB (Figure 4) enhances multi-scale feature fusion through Triple-Channel Window Attention (TCWA). In the released implementation, the input $X_{\mathrm{in}}^{l}$ at layer *l* undergoes batch normalization, channel soft splitting, three branch-specific transformations, gated restoration, a second batch normalization, and FFN refinement with residual connections.

The motivation for TCWB is that conventional single-path cross-scale fusion can blur distinct cues when high-level semantic features are merged with low-level detail. Window attention captures structured local relations efficiently, but it is constrained by the selected window partition and may miss non-windowed local patterns. Conversely, convolutional fusion preserves local continuity but has limited explicit relational modeling. TCWB addresses this trade-off by processing the same fused feature tensor through three parallel channels: a standard-window branch for positional relations inside local windows, a multi-kernel branch for continuous local structures and irregular obstacle shapes, and a frequency-aware window branch for spectral modulation before attention. The gated restoration step then recombines these representations without forcing all information through one homogeneous fusion operator.

TCWA splits the input into three channels via Channel Soft Split (SS):

**Standard Window Branch.** Features $X_{sw}^{l}$ are partitioned into non-overlapping windows. Within each window, standard multi-head attention is computed with Relative Position Bias (RPB):

(13) $${\mathrm{Attn}}_{sw}=\mathrm{Softmax}\;\left(\frac{q_{sw}\cdot k_{sw}^{T}}{\sqrt{d_{k}}}+\mathrm{RPB}\right)v_{sw}$$

**Multi-Kernel Branch.** Features $X_{l}^{l}$ are processed through depthwise convolutional branches with 3×3 and 5×5 kernels to capture local structural patterns at multiple scales without window partitioning.

**Frequency-Aware Window Branch.** Features $X_{fw}^{l}$ are first processed by FDA, then partitioned into windows with separate attention computation and RPB:

(14) $${\mathrm{Attn}}_{fw}=\mathrm{Softmax}\;\left(\frac{q_{fw}\cdot k_{fw}^{T}}{\sqrt{d_{k}}}+\mathrm{RPB}\right)v_{fw}$$

After Window Restoration, the three branches are concatenated along the channel dimension, modulated by a lightweight sigmoid gate, and projected back to the original dimensionality through a 1×1 convolution to produce the final output $X_{\mathrm{out}}^{l}$:

(15) $$X_{\mathrm{out}}^{l}={\mathrm{Conv}}_{1\times 1}\;\left(g⊙[\mathrm{WR}\left({\mathrm{Attn}}_{sw}\right);\;X_{mk}^{l};\;\mathrm{WR}\left({\mathrm{Attn}}_{fw}\right)]\right)+X_{\mathrm{in}}^{l}$$

where WR denotes Window Restoration, $X_{mk}^{l}$ is the multi-kernel branch output, and g is the channel gate computed from the concatenated branch features. The residual connection ensures stable gradient flow.

This triple-channel design ensures complementary coverage: the standard-window branch captures window-level spatial relations; the multi-kernel branch handles local structural patterns without windowing constraints, enabling it to model diverse obstacle geometries; and the frequency-aware branch provides spectral modulation before window-level attention. Compared to single-pathway fusion approaches such as BiFPN or PAFPN, TCWB processes features through three parallel and diverse pathways, allowing different types of information to be preserved rather than homogenized through sequential processing.

### 3.5. Adaptive Scale-Aware Detection Loss

Small targets are often overwhelmed by larger objects during training. We propose the Adaptive Scale-Aware Detection Loss (ASDL), detailed in Algorithm 1, which assigns scale-dependent weights to emphasize small-target supervision.

ASDL is introduced because the architectural modules alone cannot fully correct the imbalance in the training objective. In water-scene detection, small objects contribute fewer pixels and weaker gradients than larger boats, bridges, or docks, while missed small obstacles are operationally important for navigation safety. The loss therefore increases the relative contribution of matched small-object boxes in both L1 and CIoU regression terms while keeping the classification term in the RT-DETR-style matching framework. Unlike a hard small/medium/large threshold, the proposed continuous weighting function changes smoothly with relative object area, reducing abrupt optimization behavior near scale boundaries and making the same loss applicable across WaterScenes, LaRS, and IWHR. ASDL is used only during optimization: it introduces neither trainable architectural parameters nor inference-time FLOPs.

**Algorithm 1** Adaptive Scale-Aware Detection Loss (ASDL)**Require:** 
Predictions $P={\left\{\left({\hat{c}}_{i},{\hat{b}}_{i}\right)\right\}}_{i=1}^{N_{q}}$, Ground truth $G={\left\{\left(c_{j},b_{j}\right)\right\}}_{j=1}^{M}$
**Require:** 
Hyperparameters: $\lambda_{\mathrm{bbox}},\lambda_{\mathrm{ciou}},\lambda_{\mathrm{aux}},\alpha ,\beta ,\gamma$
**Ensure:** 
Total loss $L_{\mathrm{total}}$

  1:
Compute cost matrix $C_{ij}=-{\hat{p}}_{i}\left(c_{j}\right)+L_{\mathrm{box}}\left({\hat{b}}_{i},b_{j}\right)$

  2:
Find optimal assignment $\sigma^{*}=\mathrm{Hungarian}\left(C\right)$
▹ [35]
  3:
**for** each matched pair $\left(i,\sigma^{*}\left(i\right)\right)$ **do**

  4:
      $l_{\mathrm{cls}}^{i}\leftarrow -\alpha_{t}{\left(1-{\hat{p}}_{i}\right)}^{\gamma }\log\left({\hat{p}}_{i}\right)$
▹ Focal Loss [25]
  5:
      ${\mathrm{iou}}_{i}\leftarrow \mathrm{CIoU}\left({\hat{b}}_{i},b_{\sigma^{*}\left(i\right)}\right)$
▹ CIoU [31]
  6:
      $w_{i}\leftarrow {\left(1-\sqrt{A\left(b_{\sigma^{*}\left(i\right)}\right)\;/\;A\left(I\right)}\right)}^{\;\beta }$
▹ Scale-aware weight
  7:
      $l_{L1}^{i}\leftarrow {∥{\hat{b}}_{i}-b_{\sigma^{*}\left(i\right)}∥}_{1}$

  8:
      $l_{\mathrm{bbox}}^{i}\leftarrow w_{i}\cdot l_{L1}^{i}$

  9:
      $l_{\mathrm{ciou}}^{i}\leftarrow w_{i}\cdot \left(1-{\mathrm{iou}}_{i}\right)$

10:
**end for**

11:
$L_{\mathrm{cls}}\leftarrow \frac{1}{M}\sum_{i}l_{\mathrm{cls}}^{i}$,    $L_{\mathrm{bbox}}\leftarrow \frac{1}{M}\sum_{i}l_{\mathrm{bbox}}^{i}$,    $L_{\mathrm{ciou}}\leftarrow \frac{1}{M}\sum_{i}l_{\mathrm{ciou}}^{i}$

12:
$L_{\mathrm{aux}}\leftarrow L_{\mathrm{cls}}^{\mathrm{enc}}+\lambda_{\mathrm{bbox}}L_{\mathrm{bbox}}^{\mathrm{enc}}+\lambda_{\mathrm{ciou}}L_{\mathrm{ciou}}^{\mathrm{enc}}$
▹ Auxiliary encoder loss
13:
**return **
$L_{\mathrm{total}}=L_{\mathrm{cls}}+\lambda_{\mathrm{bbox}}L_{\mathrm{bbox}}+\lambda_{\mathrm{ciou}}L_{\mathrm{ciou}}+\lambda_{\mathrm{aux}}L_{\mathrm{aux}}$


The scale-aware weight $w_{i}$ is a monotonically decreasing nonlinear function of the relative object area, not an inversely proportional function. For small targets where A(B)≪A(I), $w_{i}$ approaches 1 and increases the relative regression emphasis; as object area increases, $w_{i}$ decreases smoothly. The term “adaptive” refers to this per-instance, target-scale-dependent weighting during optimization. The exponent β is a fixed global hyperparameter selected before training, rather than a dynamically learned or dynamically updated weight. The regression term combines weighted L1 localization error with weighted CIoU, matching the released implementation. The auxiliary loss $L_{\mathrm{aux}}$ supervises encoder outputs using the same classification and regression structure. We set $\lambda_{\mathrm{bbox}}=5.0$, $\lambda_{\mathrm{ciou}}=1.0$, $\lambda_{\mathrm{aux}}=1.0$, α=0.75, β=2.0, and γ=2.0 by default.

## 4. Experiments

### 4.1. Datasets and Evaluation Metrics

We evaluate WaterMamba on three challenging water scene benchmarks.

WaterScenes [33] is a large-scale multi-task dataset for water scene perception collected from diverse inland waterway environments. The official release contains multiple perception tasks and its original object labels differ from the working taxonomy used here. For the RGB-only detection experiments, we used a pre-converted detection annotation set with eight working categories: boat, buoy, swimmer, jetski, debris, bridge, dock, and animal. These working labels should therefore not be read as the original WaterScenes taxonomy. Images were acquired across multiple time slots (daytime, dusk, nighttime) and weather conditions (clear, cloudy, rainy, foggy). We follow an image-level split of 43,200 training images and 10,800 validation images. Since this split was not sequence-level, the possibility of similarity between temporally adjacent images in training and evaluation cannot be fully excluded and is acknowledged as a limitation. On average, approximately 62% of annotated instances qualify as small objects (area $<32^{2}$ pixels), making this dataset particularly suitable for evaluating small-target detection capability.

LaRS [34] is a panoptic maritime obstacle detection dataset comprising 4000 key frames collected across diverse maritime environments including harbors, open seas, rivers, and coastal areas. The official benchmark uses a 65%/5%/30% train/validation/test design with withheld test labels and server-side evaluation. Because this paper evaluates an offline object-detection protocol rather than the official panoptic benchmark, we converted the available annotated masks into tight axis-aligned bounding boxes and used a 3200/800 train/evaluation split. All retained obstacle instances are evaluated as a single obstacle class. The split was generated before training and was kept identical for all compared detectors. We do not present this result as an official LaRS test-server score. The dataset is particularly challenging due to its inclusion of direct sun glare, dense fog, and night scenes.

IWHR [36] (IWHR Floater V1) is a recently released annotated benchmark specifically focused on detecting floating debris in Chinese inland waterways. According to the public Figshare release, the dataset contains 3000 annotated images. We use an image-level 80/20 split with 2400 images for training and 600 images for testing. Because these images were collected from surveillance cameras at hydraulic monitoring stations, temporal or scene-level correlation may remain when only image-level splitting is used. The images include floating vegetation mats, plastic waste, timber logs, and mixed debris clusters captured under turbid flows, flood-season high water levels, and low-visibility conditions (see Table 1).

We adopt standard COCO-style evaluation metrics: mAP_50_ (AP at IoU = 0.5), mAP_50:95_ (AP averaged over IoU thresholds from 0.5 to 0.95), and AP_*S*_ (AP for small objects with area $<32^{2}$ pixels). Average precision is computed from confidence-ranked predictions over the full evaluation set and is therefore not determined by the single confidence threshold used to render qualitative panels. Model efficiency is measured by parameter count (Params, M) and floating-point operations (FLOPs, G). The reported results should be interpreted under this unified RGB-only detection protocol, rather than as direct copies of the original multi-task or panoptic benchmark tables.

### 4.2. Implementation Details

All experiments are implemented in PyTorch 2.1 and conducted on NVIDIA A100 GPUs. The complete model is trained end-to-end for 120 epochs using the AdamW optimizer with weight decay $10^{-4}$. The initial learning rate is set to $1\times 10^{-4}$, following a cosine annealing schedule with a five-epoch linear warmup from $10^{-6}$. The batch size is 16 and the input resolution is fixed at 640×640 pixels.

Data augmentation follows a multi-stage pipeline: (1) mosaic augmentation combining four randomly sampled images during the first 100 epochs; (2) random horizontal flipping with probability 0.5; (3) random scaling in the range [0.5,1.5]; (4) color jitter with brightness, contrast, and saturation perturbations of ±0.4; and (5) random cropping to the target resolution. In the final 20 epochs, mosaic is disabled to allow the model to adapt to single-image inference conditions, following the training strategy of YOLOX [37].

The number of object queries in the decoder is set to 300. In the released MRFFI implementation, the multi-range branches use depthwise convolutions with kernel sizes 3, 5, and 7, followed by a fixed Haar-style rearrangement path and bidirectional selective scan. The window size in TCWB is 8×8. For the ASDL loss, we set $\lambda_{\mathrm{bbox}}=5.0$, $\lambda_{\mathrm{ciou}}=1.0$, $\lambda_{\mathrm{aux}}=1.0$, α=0.75, γ=2.0, and β=2.0. Hyperparameters were selected in a preliminary WaterScenes validation run and then held fixed for LaRS and IWHR. The reported WaterScenes validation results are not a separately nested test after model selection, so model-selection bias cannot be fully excluded.

For baseline reproducibility, all compared detectors were trained or fine-tuned with the same image resolution, train/test splits, data augmentations, optimizer family, and evaluation script whenever their official implementations allowed it. Pre-trained weights were used only for backbone initialization, and no method was evaluated using test-set tuning. The values in Table 2, Table 3, Table 4, Table 5 and Table 6 are therefore internally comparable within our protocol. They may differ from values reported in the original dataset papers because those papers use different task definitions, label mappings, modalities, or evaluation settings. The public source repository contains the model modules, dataset readers, training, evaluation, and inference entry points, together with configurations for WaterScenes, LaRS, and IWHR; repository timestamps document package maintenance rather than the execution time of private/local experiments.

### 4.3. Comparison with State-of-the-Art on WaterScenes

Table 2 presents the comparison on the WaterScenes dataset. We include methods from two-stage, one-stage, and transformer-based paradigms to show where WaterMamba sits among common detector families. To avoid overemphasizing the number of baselines, the discussion below focuses on representative and reproducible comparisons, especially RT-DETR-L, YOLOv13, and CO-DETR.

**Table 2:** Comparison with state-of-the-art methods on the WaterScenes dataset. Best results in **bold**, second best underlined.

Method	mAP_50_	mAP_50:95_	AP_*S*_	Params (M)	FLOPs (G)
Two-Stage Detectors					
Faster R-CNN [4]	62.3	35.8	22.1	41.1	134.4
Cascade R-CNN [22]	65.1	38.4	24.6	69.2	189.6
Dynamic R-CNN [23]	63.8	37.1	23.4	41.1	134.4
Sparse R-CNN [24]	64.5	37.9	23.8	106.1	166.3
One-Stage Detectors					
RetinaNet [25]	61.2	34.6	20.8	36.3	152.5
FCOS [26]	62.8	36.2	21.5	32.1	128.7
YOLOX-L [37]	68.5	41.2	27.1	54.2	155.6
YOLOv5-L [5]	67.4	40.1	26.3	46.5	109.1
YOLOv7 [6]	69.2	41.5	27.4	36.9	104.7
YOLOv8-L [38]	70.6	42.8	28.7	43.7	165.2
YOLOv9-C [7]	71.3	43.2	29.1	25.3	102.8
YOLOv10-L [8]	72.1	44.0	30.2	24.4	120.3
YOLOv11-L [39]	72.8	44.6	30.8	25.3	86.9
YOLOv12-L [9]	73.5	45.3	31.5	26.4	88.6
YOLOv13 [10]	74.2	45.8	32.0	29.1	93.4
RTMDet-L [40]	71.5	43.4	29.3	52.3	204.8
Transformer-Based Detectors					
DETR [11]	60.4	33.8	19.2	41.3	86.0
Deformable DETR [12]	66.7	39.5	25.8	40.0	173.0
DAB-DETR [28]	67.5	40.2	26.4	43.7	194.0
DINO [13]	71.8	43.6	29.5	47.0	279.0
RT-DETR-L [14]	74.5	45.9	32.3	32.0	108.0
CO-DETR [30]	73.2	44.7	30.6	47.2	293.0
**WaterMamba (Ours)**	**77.3**	**48.6**	**36.8**	28.6	96.2

WaterMamba achieves 77.3% mAP_50_ and 48.6% mAP_50:95_ under our unified detection protocol. Compared with RT-DETR-L, WaterMamba improves mAP_50_ by 2.8 percentage points and AP_*S*_ by 4.5 points. The larger AP_*S*_ difference is consistent with the intended emphasis on small targets, while the contribution of individual components is assessed separately in Section 4.6. Compared with YOLOv13, WaterMamba achieves a 3.1-point higher mAP_50_ with slightly fewer parameters (28.6 M vs. 29.1 M), but slightly higher FLOPs (96.2 G vs. 93.4 G). This comparison therefore supports an accuracy–parameter trade-off rather than an across-the-board computational advantage.

We note that some baseline values differ from those reported in the original WaterScenes publication. This difference arises because the present study evaluates RGB-only object detection after label conversion and unified retraining, whereas the original benchmark reports results under its own multi-task dataset protocol. The results in Table 2 should therefore be used for within-paper comparison under identical settings, not as a replacement for the original WaterScenes benchmark table.

Table 3 further analyzes per-category AP_50_ on WaterScenes for the top-performing methods. WaterMamba achieves the best performance on seven of eight categories, with differences of 5.2 points for Buoy, 4.8 points for Swimmer, and 6.1 points for Debris relative to RT-DETR-L. These categories frequently include distant or partially submerged objects against water-surface clutter. The category-level pattern is consistent with the design objective, although causal attribution to FDA is based on the controlled component ablation rather than this comparison alone.

**Table 3:** Per-category AP_50_ (%) comparison on the WaterScenes dataset for top-performing methods.

Method	Boat	Buoy	Swim.	Jetski	Debris	Bridge	Dock	Animal	Mean
RT-DETR-L	82.1	65.3	58.7	80.5	52.4	89.2	85.6	82.2	74.5
YOLOv13	81.6	64.8	57.2	79.8	51.6	88.5	85.2	84.9	74.2
YOLOv12-L	80.8	63.5	56.4	78.9	50.8	87.8	84.6	85.2	73.5
CO-DETR	80.2	62.1	55.8	78.2	49.5	87.3	84.1	88.4	73.2
DINO	78.5	60.7	54.1	77.0	48.2	86.1	82.8	87.0	71.8
**WaterMamba**	**84.3**	**70.5**	**63.5**	**82.7**	**58.5**	**90.8**	**87.4**	80.7	**77.3**

Note: Bold indicates the best result in each column.

To examine whether the improvement is concentrated only in easy scenes, Table 4 provides a coarse condition-level analysis on the WaterScenes evaluation split where metadata or manual tags are available. WaterMamba improves over RT-DETR-L in all four subsets, with larger gains in low-light and degraded-visibility cases than in clear daytime scenes. This pattern is consistent with the design goal of combining long-range spatial context, spectral modulation, and scale-aware optimization. Because these subsets are not sequence-independent test sets, the table is used as an auxiliary robustness analysis rather than a separate benchmark.

**Table 4:** Condition-level mAP_50_ (%) analysis on the WaterScenes evaluation split. Values are grouped by coarse scene condition and should be interpreted as an auxiliary analysis under the same image-level protocol.

Condition Subset	Images	RT-DETR-L	YOLOv13	WaterMamba
Clear/daytime	6200	78.9	78.4	**81.1**
Dusk/low-light	1800	72.6	72.1	**75.5**
Rain/fog	1400	69.3	68.7	**72.8**
Glare/turbid water	1400	68.1	67.4	**71.4**

Note: Bold indicates the best result in each row.

We also evaluate inference speed on an NVIDIA A100 GPU with input resolution 640×640. WaterMamba achieves 78.4 FPS (frames per second), compared with 114.2 FPS for RT-DETR-L, 87.6 FPS for YOLOv13, and 45.2 FPS for CO-DETR. Relative to RT-DETR-L, WaterMamba reduces FLOPs by 10.9% and parameters by 10.6%, but its measured throughput is 31.3% lower. FLOPs and parameter counts do not capture operator-level efficiency, memory traffic, kernel-launch overhead, or achievable parallelism. In particular, FDA adds FFT/IFFT operations whose server-GPU latency is not reflected adequately by aggregate FLOPs. WaterMamba is therefore slower than RT-DETR-L and YOLOv13 on this GPU, but faster than DINO (32.1 FPS) and CO-DETR. These measurements characterize the accuracy–throughput trade-off on an A100 and should not be interpreted as evidence of onboard ASV latency.

**Figure 5:**
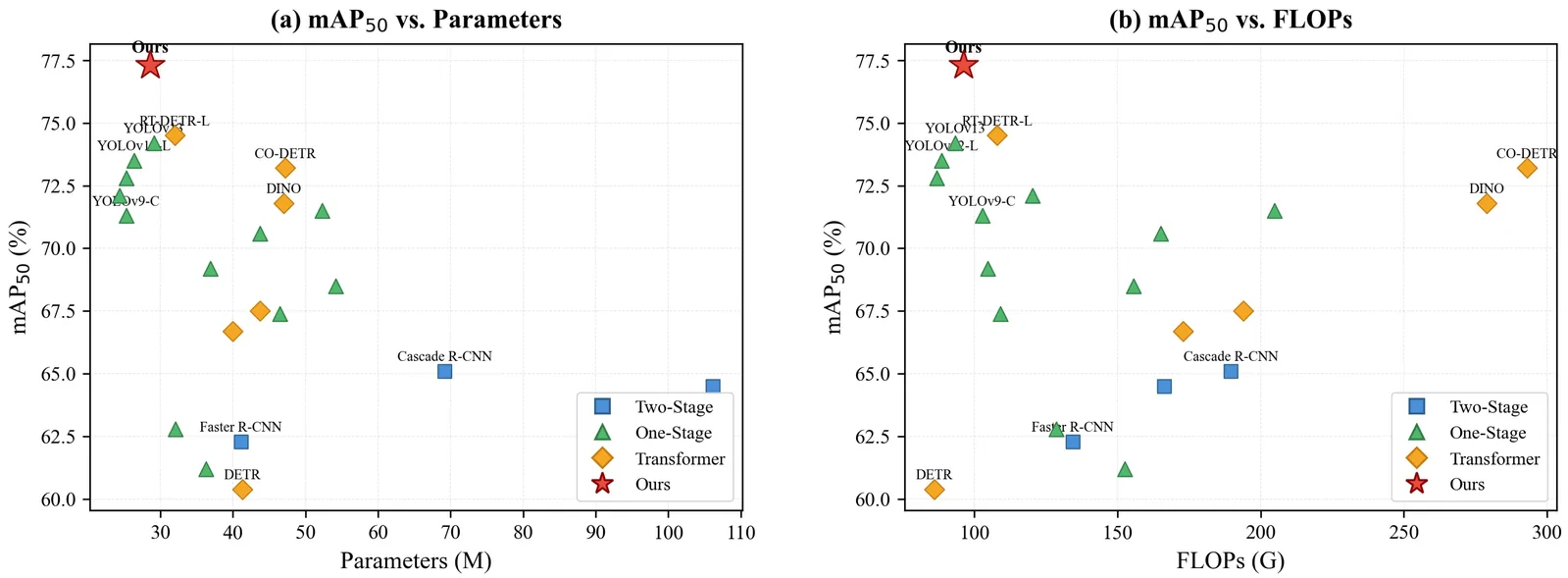
Accuracy-efficiency trade-off analysis on the WaterScenes dataset. (**a**) mAP_50_ vs. model parameters. (**b**) mAP_50_ vs. FLOPs. WaterMamba (red star) achieves the highest accuracy and combines this result with a competitive parameter count in panel (**a**). In panel (**b**), several YOLO variants lie further left because they require fewer FLOPs, whereas WaterMamba provides higher accuracy at 96.2 G FLOPs.

To further illustrate the accuracy-efficiency trade-off, Figure 5 visualizes mAP_50_ against model parameters and FLOPs for all compared methods on WaterScenes. WaterMamba achieves the highest accuracy with moderate computational cost. Notably, our model surpasses all transformer-based competitors (including CO-DETR with 293 G FLOPs) while requiring 96.2 G FLOPs, and it outperforms all one-stage detectors despite having fewer parameters than most of them.

### 4.4. Comparison with State-of-the-Art on LaRS

Table 5 shows results on the LaRS maritime dataset. WaterMamba consistently outperforms all methods.

On LaRS, WaterMamba achieves 73.6% mAP_50_, 2.8 points above RT-DETR-L and 3.1 points above YOLOv13. Its AP_*S*_ is 4.8 points above RT-DETR-L. Because the architecture and hyperparameters are held fixed across datasets, this result supports cross-dataset consistency under the present protocol. The LaRS result alone does not isolate FDA; that attribution is evaluated in the WaterScenes component ablation.

**Table 5:** Comparison with state-of-the-art methods on the LaRS dataset. Best in **bold**, second best underlined.

Method	mAP_50_	mAP_50:95_	AP_*S*_	Params (M)	FLOPs (G)
Two-Stage Detectors					
Faster R-CNN [4]	58.6	32.4	18.7	41.1	134.4
Cascade R-CNN [22]	61.3	35.1	21.2	69.2	189.6
Dynamic R-CNN [23]	59.8	33.7	19.9	41.1	134.4
Sparse R-CNN [24]	60.5	34.3	20.4	106.1	166.3
One-Stage Detectors					
RetinaNet [25]	57.4	31.2	17.5	36.3	152.5
FCOS [26]	59.1	33.0	18.3	32.1	128.7
YOLOX-L [37]	64.8	38.0	23.8	54.2	155.6
YOLOv5-L [5]	63.5	36.8	22.7	46.5	109.1
YOLOv7 [6]	65.4	38.6	24.1	36.9	104.7
YOLOv8-L [38]	66.9	39.8	25.3	43.7	165.2
YOLOv9-C [7]	67.5	40.3	25.8	25.3	102.8
YOLOv10-L [8]	68.3	41.1	26.5	24.4	120.3
YOLOv11-L [39]	69.0	41.7	27.1	25.3	86.9
YOLOv12-L [9]	69.8	42.4	27.8	26.4	88.6
YOLOv13 [10]	70.5	43.0	28.4	29.1	93.4
RTMDet-L [40]	67.8	40.5	25.6	52.3	204.8
Transformer-Based Detectors					
DETR [11]	56.8	30.5	16.3	41.3	86.0
Deformable DETR [12]	63.0	36.2	22.1	40.0	173.0
DAB-DETR [28]	63.8	37.0	22.8	43.7	194.0
DINO [13]	68.0	40.7	26.0	47.0	279.0
RT-DETR-L [14]	70.8	43.1	28.7	32.0	108.0
CO-DETR [30]	69.5	41.8	27.0	47.2	293.0
**WaterMamba (Ours)**	**73.6**	**45.8**	**33.5**	28.6	96.2

### 4.5. Comparison with State-of-the-Art on IWHR

Table 6 presents the comparison on the IWHR inland waterway dataset.

On IWHR, WaterMamba achieves 71.5% mAP_50_ and 44.2% mAP_50:95_. Its AP_*S*_ of 32.1% is 4.8 points above RT-DETR-L. This full-set difference is consistent with improved sensitivity to small floating hazards under the stated protocol. IWHR includes floating vegetation and debris against turbid water, but the benchmark-level comparison is not used by itself to claim a specific spectral mechanism.

**Figure 6:**
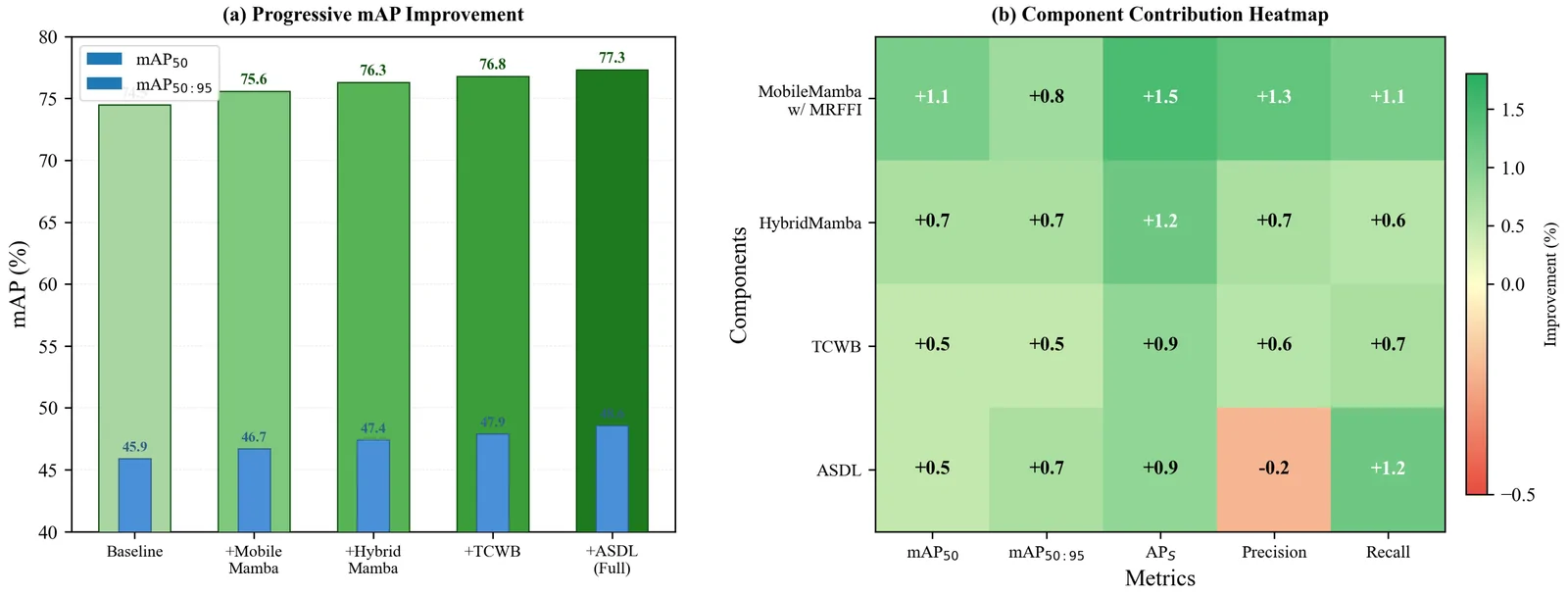
Detailed analysis of module contributions on WaterScenes. (**a**) Progressive mAP improvement with gradient-coded bars showing mAP_50_ (green, outer) and mAP_50:95_ (blue, inner) as architecture and optimization changes are progressively incorporated. (**b**) Component contribution heatmap showing individual improvement (Δ%) of each module across five evaluation metrics; the ASDL precision change is −0.2 percentage points.

To provide a more fine-grained understanding of each module’s contribution, Figure 6 presents the progressive mAP improvement alongside a component contribution heatmap. As shown in Figure 6a, the green-shaded bars illustrate the steady mAP_50_ growth from 74.5% (baseline) to 77.3% (full model), while the inner blue bars track mAP_50:95_ improvements. The heatmap in Figure 6b reveals that MobileMamba with MRFFI yields the largest individual gains across most metrics, particularly +1.5% on AP_*S*_. Notably, ASDL shows a slight precision decrease (−0.2%) but substantially boosts recall (+1.2%), consistent with its design to increase sensitivity toward hard-to-detect small targets.

**Table 6:** Comparison with state-of-the-art methods on the IWHR dataset. Best in **bold**, second best underlined.

Method	mAP_50_	mAP_50:95_	AP_*S*_	Params (M)	FLOPs (G)
Two-Stage Detectors					
Faster R-CNN [4]	56.2	30.8	17.3	41.1	134.4
Cascade R-CNN [22]	59.1	33.5	19.8	69.2	189.6
Dynamic R-CNN [23]	57.5	32.1	18.5	41.1	134.4
Sparse R-CNN [24]	58.3	32.8	19.0	106.1	166.3
One-Stage Detectors					
RetinaNet [25]	55.0	29.6	16.1	36.3	152.5
FCOS [26]	56.8	31.4	16.9	32.1	128.7
YOLOX-L [37]	62.5	36.4	22.4	54.2	155.6
YOLOv5-L [5]	61.3	35.2	21.3	46.5	109.1
YOLOv7 [6]	63.1	37.0	22.7	36.9	104.7
YOLOv8-L [38]	64.6	38.2	23.9	43.7	165.2
YOLOv9-C [7]	65.2	38.7	24.4	25.3	102.8
YOLOv10-L [8]	66.0	39.5	25.1	24.4	120.3
YOLOv11-L [39]	66.7	40.1	25.7	25.3	86.9
YOLOv12-L [9]	67.5	40.8	26.4	26.4	88.6
YOLOv13 [10]	68.2	41.4	27.0	29.1	93.4
RTMDet-L [40]	65.5	38.9	24.2	52.3	204.8
Transformer-Based Detectors					
DETR [11]	54.4	28.9	14.9	41.3	86.0
Deformable DETR [12]	60.7	34.6	20.7	40.0	173.0
DAB-DETR [28]	61.5	35.4	21.4	43.7	194.0
DINO [13]	65.7	39.1	24.6	47.0	279.0
RT-DETR-L [14]	68.5	41.5	27.3	32.0	108.0
CO-DETR [30]	67.2	40.2	25.6	47.2	293.0
**WaterMamba (Ours)**	**71.5**	**44.2**	**32.1**	28.6	96.2

### 4.6. Ablation Study

We conduct comprehensive ablation studies on WaterScenes to validate each component’s contribution. Table 7 reports progressive performance results. The configurations are sequential replacements of the corresponding RT-DETR-L backbone, encoder, and cross-scale refinement operations, rather than a purely additive stack. ASDL is optimization-only and does not change either parameter count or inference-time FLOPs.

Each component yields consistent improvements. Replacing the backbone with MobileMamba + MRFFI provides +1.1% mAP_50_, while HybridMamba further improves performance by +0.7% through spatial-spectral encoding. The replacement of the baseline cross-scale refinement by TCWB adds +0.5% through enhanced fusion. ASDL contributes +0.5% mAP_50_ and notably +0.9% AP_*S*_, confirming its effectiveness for small-target optimization. The cumulative improvement reaches +2.8% mAP_50_ and +4.5% AP_*S*_.

To complement the progressive study, Table 8 reports deletion-based ablations from the full WaterMamba configuration. This analysis is useful because the progressive sequence may understate later changes after earlier replacements have improved the representation. Removing MobileMamba+MRFFI produces the largest drop in mAP_50_ and AP_*S*_, showing that efficient multi-scale state-space feature extraction is the strongest single architectural factor. Removing HybridMamba or FDA mainly reduces AP_*S*_, indicating that spatial-spectral encoding is most relevant to small and low-contrast obstacles. Removing TCWB weakens both mAP_50:95_ and AP_*S*_, which is consistent with its role in cross-scale fusion. Removing ASDL leaves the architecture unchanged but reduces AP_*S*_ by 0.9 points, supporting the claim that scale-aware supervision contributes beyond feature design.

Table 9 isolates the contributions of HybridMamba’s submodules.

VSSM alone (row 1) provides a strong spatial baseline. Each additional submodule contributes complementary improvements: FDA provides the largest individual gain (+0.6% mAP_50_, +0.9% AP_*S*_), supporting the usefulness of frequency-domain modulation for the evaluated water scenes. MRFB and MBHA contribute multi-range and multi-branch spatial enhancement respectively, and their combination with FDA yields the best performance. These results indicate that HybridMamba’s benefit comes from combining spatial state-space modeling, multi-range local detail, branch-wise attention, and spectral modulation rather than from a single isolated operation.

**Table 9:** Component analysis of HybridMamba on WaterScenes.

VSSM	MRFB	MBHA	FDA	mAP_50_	mAP_50:95_	AP_*S*_
✔				75.3	46.4	33.5
✔	✔			75.8	46.8	34.1
✔		✔		75.7	46.7	34.0
✔			✔	75.9	46.9	34.4
✔	✔	✔		76.0	47.1	34.6
✔	✔		✔	76.1	47.2	34.8
✔	✔	✔	✔	**76.3**	**47.4**	**35.0**

Note: Bold indicates the best performance value in each metric column.

**Figure 7:**
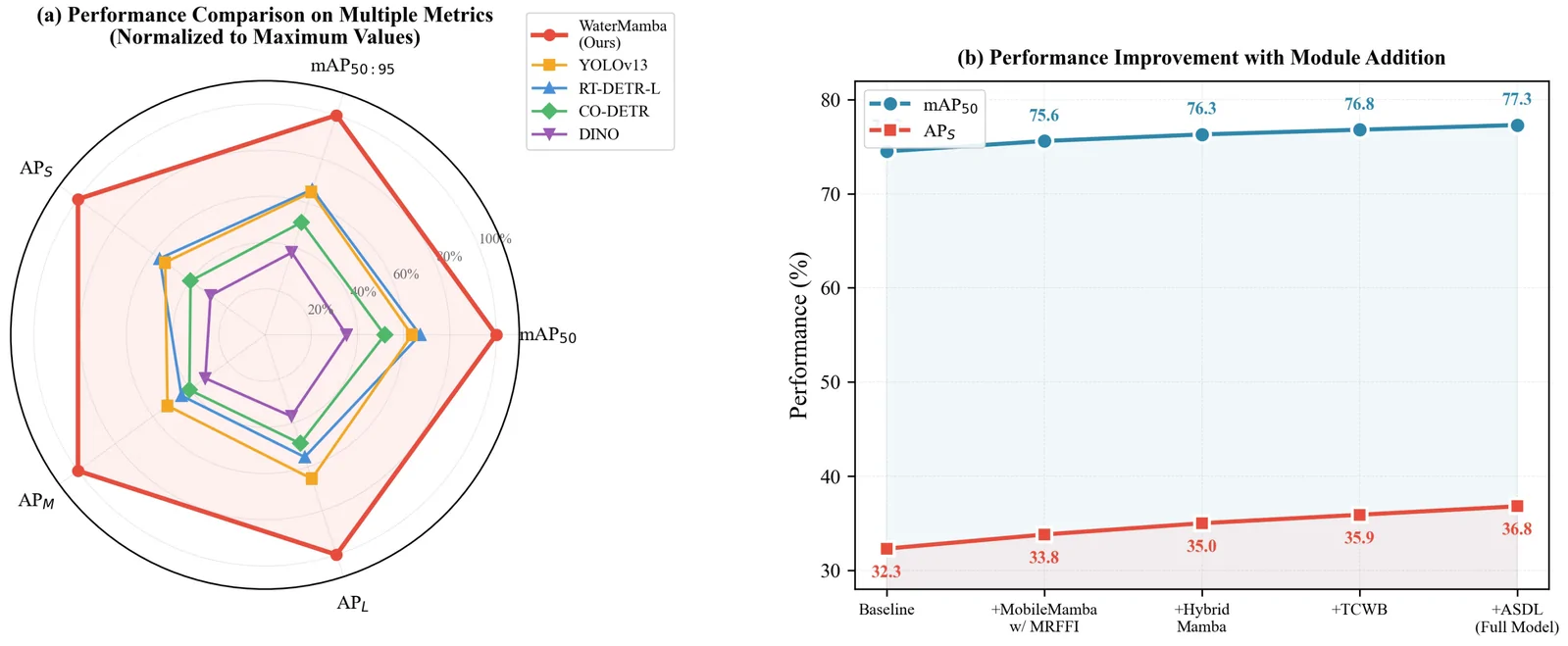
Multi-dimensional performance analysis on the WaterScenes dataset. (**a**) Radar chart comparing WaterMamba against four top-performing competitors across five evaluation metrics (normalized to maximum values). WaterMamba (red) encloses the largest area among the compared methods. (**b**) Progressive performance trend as architecture and optimization changes are incorporated, showing consistent improvement in both mAP_50_ and AP_*S*_.

Figure 7 provides multi-dimensional visualization of the results. The radar chart in Figure 7a compares WaterMamba against four top-performing competitors across five evaluation metrics normalized to their maximum values; WaterMamba (red polygon) covers the largest area among the compared methods. The progressive line chart in Figure 7b tracks the mAP_50_ and AP_*S*_ trends as architecture and optimization changes are progressively incorporated, showing consistent and steady performance gains from 74.5%/32.3% (baseline) to 77.3%/36.8% (full model).

### 4.7. Fixed-Threshold Qualitative Comparison

**Figure 8:**
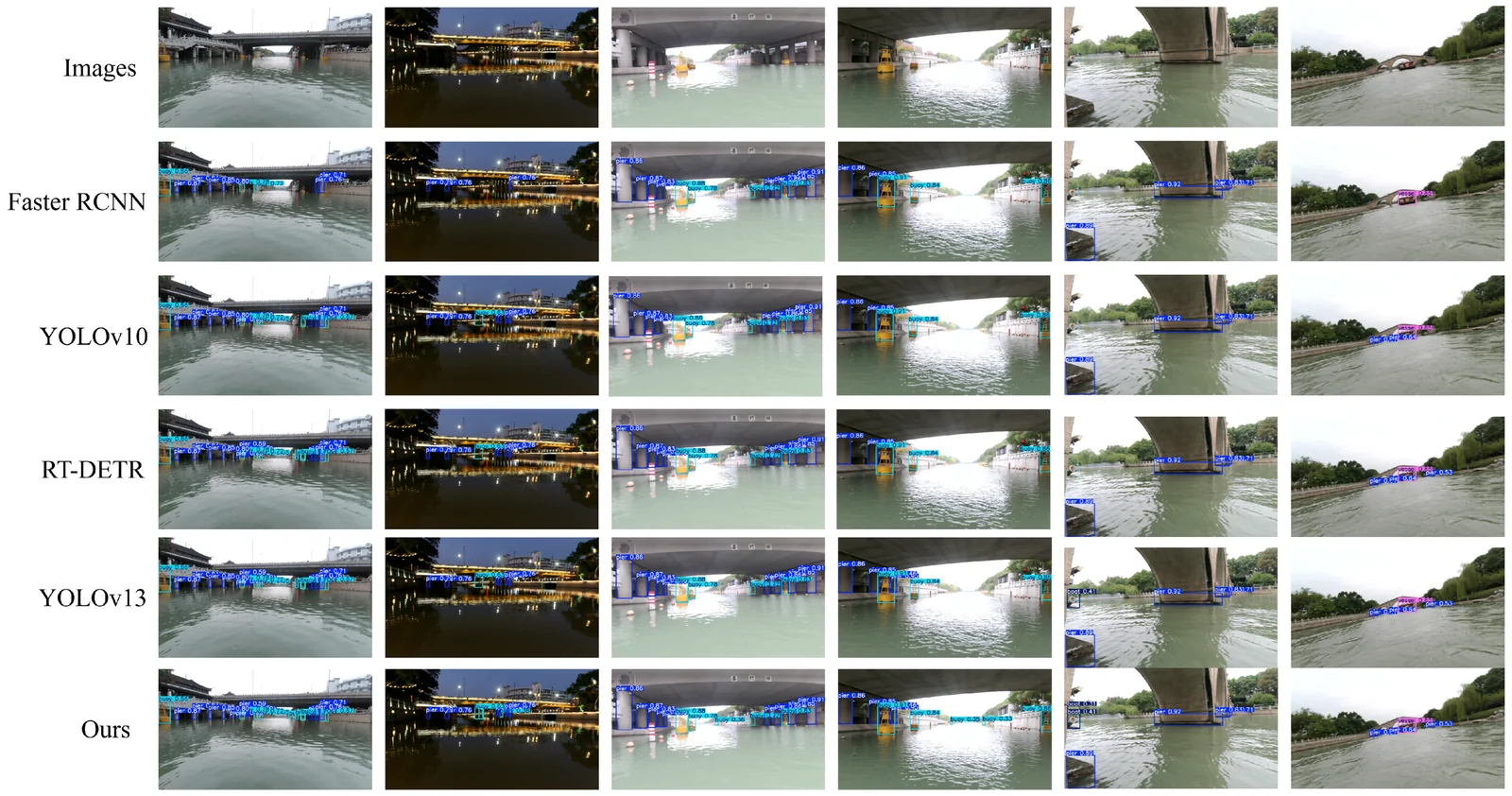
Fixed-threshold outputs on WaterScenes. All displayed predictions were generated at confidence ≥0.3. Similar boxes for jointly detected objects indicate comparable localization of shared, easier instances; the relevant qualitative contrast is the retention of additional difficult small or low-contrast targets at the same operating point. Dataset-wide performance is quantified in Table 2.

**Figure 9:**
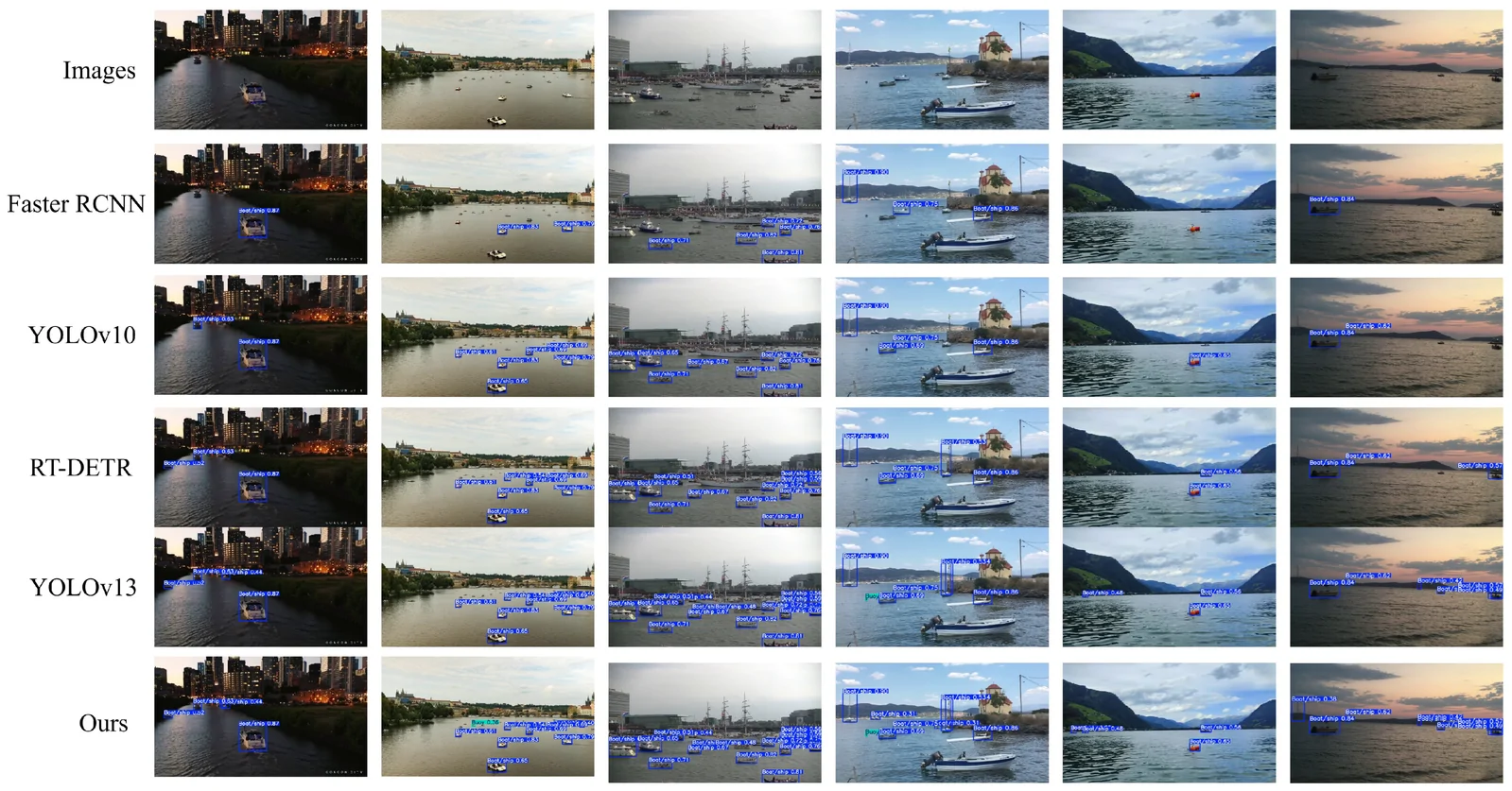
Fixed-threshold outputs on LaRS. All displayed predictions were generated at confidence ≥0.3. The scenes show the common-operating-point behavior for distant maritime obstacles under low light, glare, and complex backgrounds; the corresponding full-set comparison is reported in Table 5.

**Figure 10:**
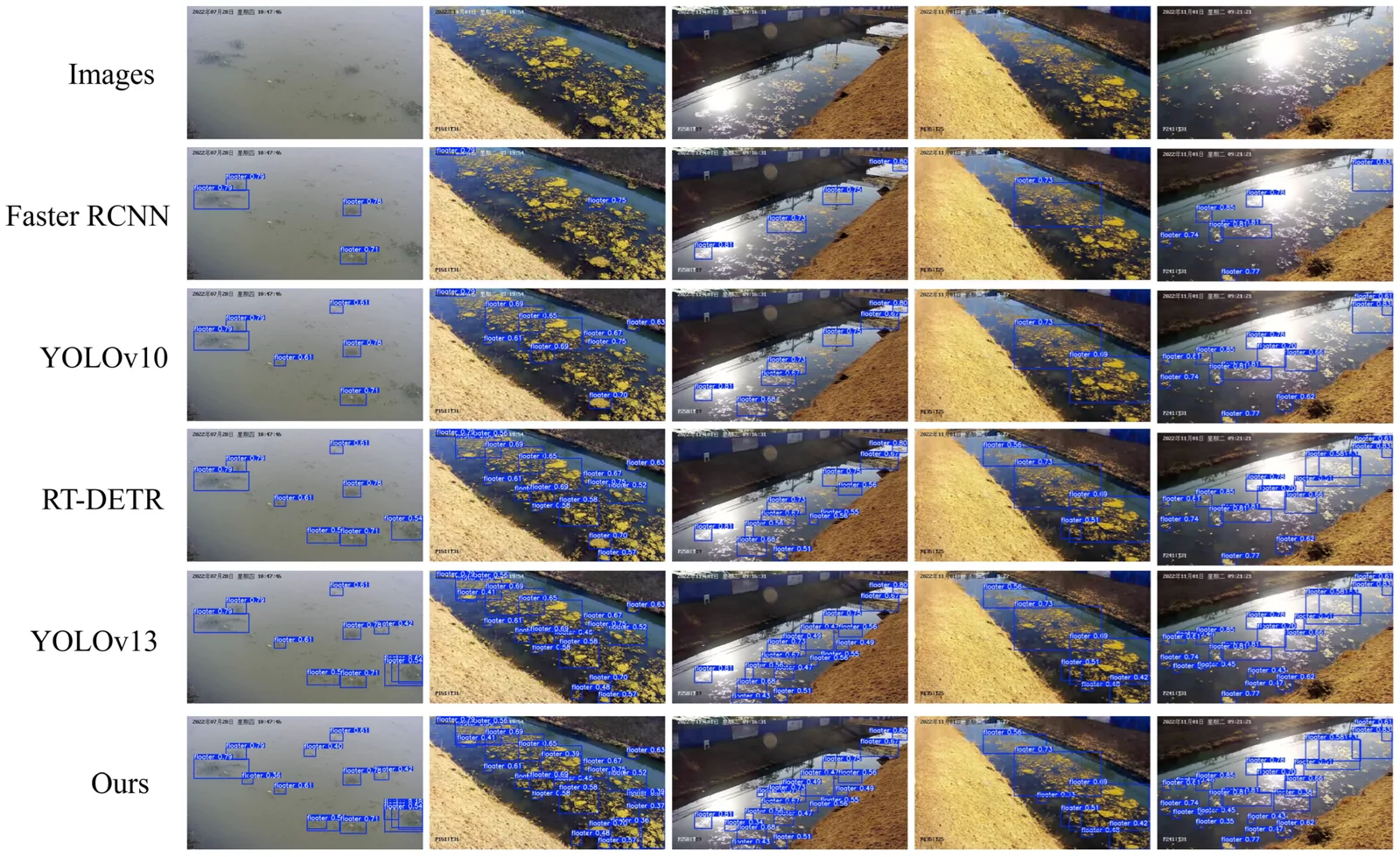
Fixed-threshold outputs on IWHR. All displayed predictions were generated at confidence ≥0.3. The scenes show the common-operating-point behavior for floating vegetation and debris in turbid waterways; the corresponding full-set comparison is reported in Table 6. The non-English text at the top of the source frames is original camera date/time metadata and is not used in the analysis.

Figure 8, Figure 9 and Figure 10 provide a fixed-operating-point comparison that complements the benchmark tables. Each panel was generated by independently applying the corresponding trained detector to the same image at the same input resolution and retaining predictions with confidence ≥0.3. No image-specific threshold selection or threshold sweep was used. The identical display rule is important: it prevents a method from obtaining a visual advantage through a more permissive threshold. The selected models cover two-stage, one-stage, and transformer-based detector families.

For objects detected by several strong models, similar coordinates, sizes, labels, and confidence scores are expected: all detectors are evaluated against the same object geometry, and the proposed advantage does not require WaterMamba to move an already correct box. Similarity among shared detections therefore does not imply the absence of a qualitative advantage. The relevant visual difference for this small-target task is whether difficult objects are retained at the same operating point. Predictions necessarily fall on one side or the other of any fixed decision threshold; describing an additional detection as a “threshold crossing” does not determine whether it is arbitrary or reflects a better learned ranking. That question is resolved at dataset scale by confidence-ranked AP, which integrates predictions beyond the single display threshold. WaterMamba’s higher mAP_50_ and AP_*S*_ across the full evaluation sets therefore rule out the interpretation that the reported advantage is created only by choosing 0.3 for visualization. The panels provide representative, supportive evidence of where the measured recall advantage appears, while Table 2, Table 3, Table 4, Table 5 and Table 6 provide the quantitative test.

Across the displayed scenes, all strong detectors localize many of the same salient objects, which explains their similar boxes and scores. This agreement is not contrary to the claimed advantage: WaterMamba’s improvement is concentrated on difficult small or low-contrast instances that competing models omit at the same threshold, rather than on producing visibly different boxes for objects that every model already detects. The panels also expose shared limitations: objects occupying only a few pixels may still be missed, while strong reflections, wakes, or vegetation texture may produce ambiguous low-confidence predictions. We do not use these examples to claim confidence calibration or universally better localization. Instead, they are qualitatively consistent with the higher full-set AP_*S*_ and show both the operating-point recall difference and the remaining failure cases.

## 5. Discussion

The experimental results indicate that WaterMamba performs consistently across all three benchmarks under the evaluation protocol used in this study. The interpretation below distinguishes benchmark-wide evidence, component-level evidence, and selected qualitative observations.

**Why WaterMamba Outperforms Competing Detectors.** The performance advantage is not attributed to a single module, but to the alignment between the architecture and the main error sources in water-scene detection. Compared with one-stage detectors, WaterMamba retains RT-DETR’s end-to-end query formulation, which reduces dependence on dense anchors and post-processing thresholds. Compared with the RT-DETR-L baseline, MobileMamba+MRFFI improves multi-scale feature extraction while reducing parameters, HybridMamba adds long-range state-space modeling and learned spectral modulation at the semantic encoder stage, TCWB preserves complementary cross-scale information during fusion, and ASDL increases the optimization weight of small matched boxes. These design choices target different parts of the detection pipeline: backbone representation, encoder context modeling, multi-scale fusion, and training supervision. This explains why the largest gains appear in AP_*S*_ and in categories such as Buoy, Swimmer, and Debris, which are typically small, low contrast, or partially confused with water-surface texture. The deletion ablation in Table 8 further shows that removing any one component reduces performance, indicating that the final result depends on integration rather than on an untested stacking of unrelated modules.

**Effectiveness and Boundary of Frequency-Domain Analysis.** The most direct evidence for FDA is the controlled HybridMamba ablation in Table 9, where adding FDA to VSSM changes mAP_50_ from 75.3% to 75.9% and AP_*S*_ from 33.5% to 34.4%. This result supports the usefulness of learned spectral modulation under the WaterScenes protocol. It does not establish that every reflection is low-frequency, every ripple is high-frequency, or spatial methods cannot represent these patterns. We therefore interpret FDA as a complementary learned transformation whose benefit is empirical and dataset-bounded.

**Per-Category Analysis.** The per-category results in Table 3 reveal a clear strength and a clear limitation. Relative to RT-DETR-L, WaterMamba improves Buoy by 5.2 points, Swimmer by 4.8 points, and Debris by 6.1 points. However, its Animal AP of 80.7 is the lowest among all methods in the table: it is 1.5 points below RT-DETR-L, 4.2 below YOLOv13, 4.5 below YOLOv12-L, 7.7 below CO-DETR, and 6.3 below DINO. The present category-level comparison does not isolate the cause of this weakness and does not establish whether it is related to FDA. Future work should examine animal-specific error patterns and whether category-aware spectral modulation can improve this failure case without reducing performance on small water-surface targets.

**Efficiency of SSM-Based Architecture.** WaterMamba (28.6 M parameters, 96.2 G FLOPs) is smaller than the RT-DETR-L baseline (32.0 M, 108.0 G), although it is slightly more expensive than YOLOv13 in FLOPs. This reduction reflects the adopted MobileMamba backbone, including the established MRFFI design, rather than a new MRFFI contribution. Importantly, the lower theoretical compute does not translate into higher throughput: 78.4 FPS is 31.3% below RT-DETR-L’s 114.2 FPS despite 10.9% fewer FLOPs. Aggregate FLOPs omit operator mix, memory movement, kernel-launch costs, and hardware utilization; the FFT/IFFT operations in FDA add latency that is not predicted well by the FLOP total. The measured throughput therefore characterizes an accuracy–latency trade-off on an NVIDIA A100 and does not establish onboard real-time feasibility. Direct deployment claims require embedded-hardware latency, memory, and power measurements.

**Figure 11:**
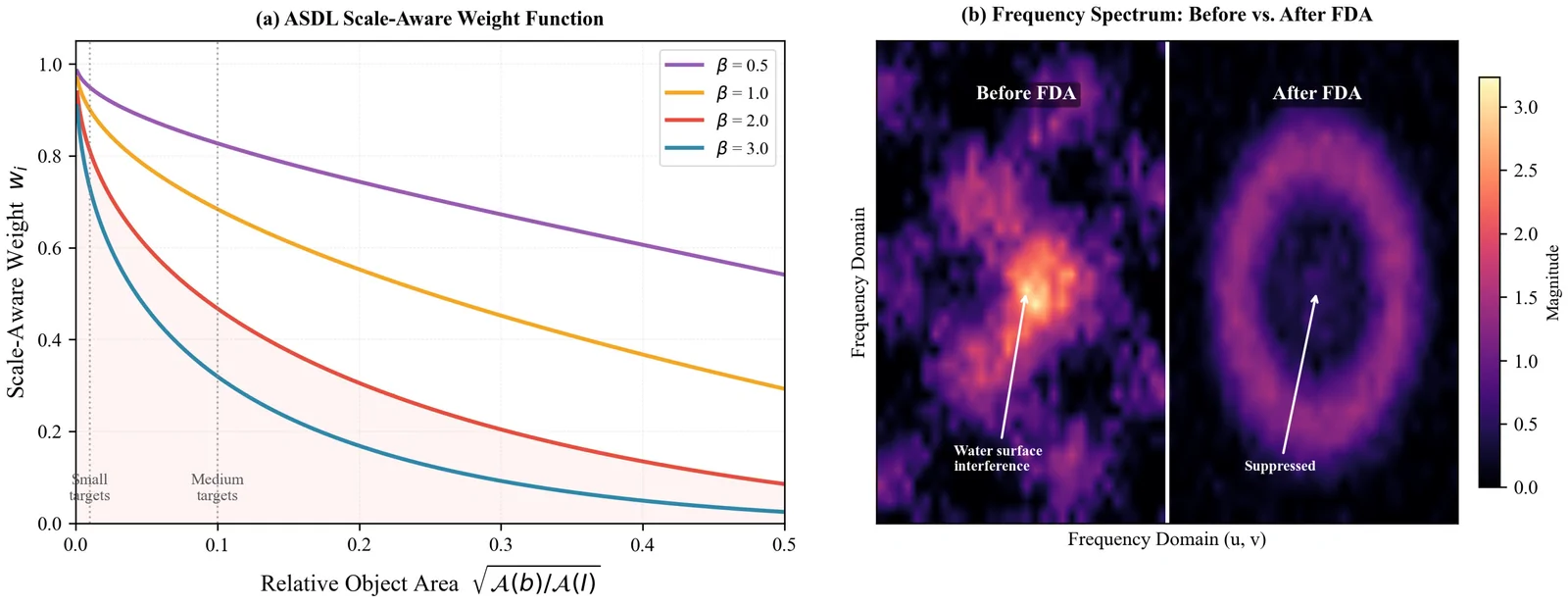
Analytical visualization of key design choices. (**a**) ASDL scale-aware weight function $w_{i}={\left(1-\sqrt{r}\right)}^{\beta }$ for varying β values, illustrating how smaller targets receive larger regression weights. (**b**) Simulated frequency-domain magnitude spectrum before (left) and after (right) spectral modulation. This panel is an explanatory schematic and is not used as empirical evidence of learned FDA behavior.

**Scale-Aware Training Strategy.** The ASDL loss contributes +0.9% AP_*S*_ in the ablation study, a proportionally larger gain than its mAP_50_ improvement (+0.5%). This asymmetric improvement pattern supports the intended role of scale-dependent weighting in emphasizing small targets. The scale-aware weight $w_{i}={\left(1-\sqrt{A(b)/A(I)}\right)}^{\beta }$ provides a smooth, differentiable reweighting that avoids hard thresholding. Figure 11a illustrates how the weight function behaves under different β values. With our default β=2.0 (red curve), small targets ($\sqrt{A/A(I)}<0.05$) receive weights exceeding 0.85, while medium-to-large targets receive progressively reduced weights. Figure 11b is a simulated illustration of spectral modulation and should be interpreted only as an explanatory schematic, not as direct empirical evidence of learned frequency behavior.

**Figure 12:**
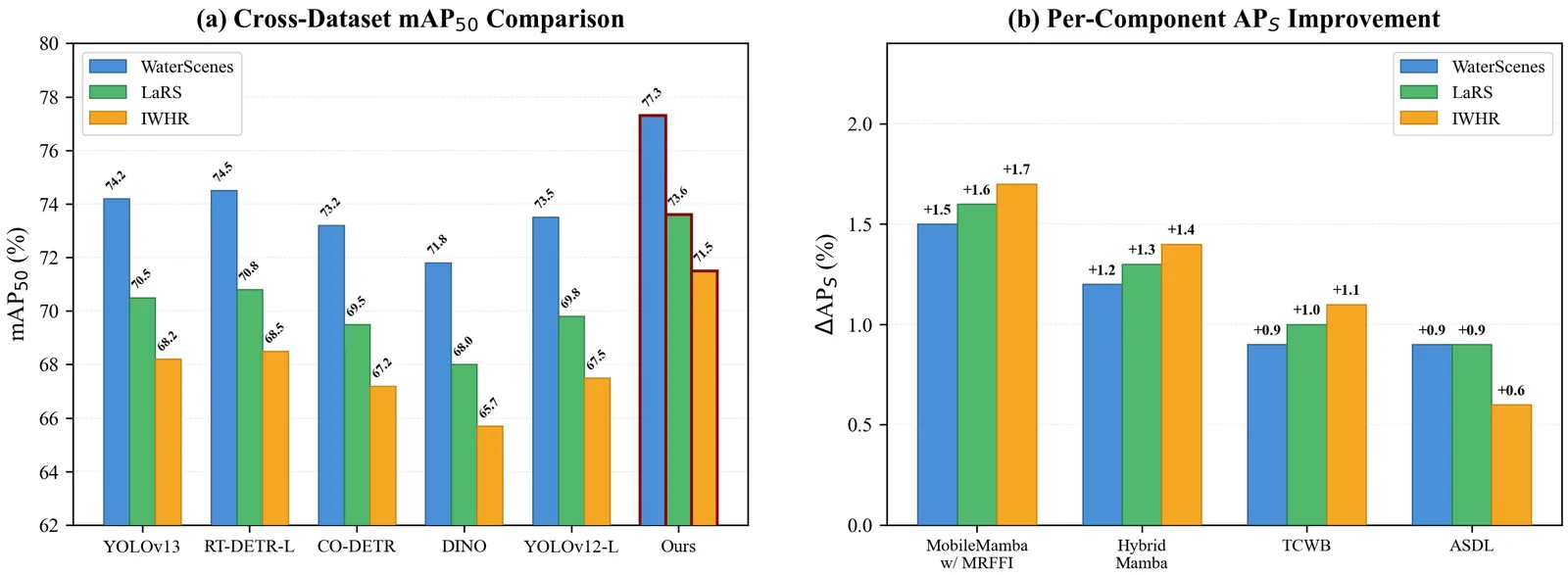
Cross-dataset performance analysis. (**a**) mAP_50_ comparison of top-performing methods across WaterScenes, LaRS, and IWHR; the red frames identify the WaterMamba (Ours) bars. (**b**) Per-component AP_*S*_ improvement (ΔAP_*S*_) contributed by each proposed module across three datasets.

**Cross-Dataset and Condition-Level Consistency.**Figure 12 summarizes the three benchmark tables. WaterMamba ranks first among the evaluated methods on each dataset under the unified protocol. Holding the main training choices fixed across datasets reduces the risk that the reported pattern is caused by dataset-specific retuning. Table 4 adds a complementary condition-level view on WaterScenes, where WaterMamba remains ahead of RT-DETR-L and YOLOv13 in clear/daytime, dusk/low-light, rain/fog, and glare/turbid subsets. The per-component bars in Figure 12b are summary values from the same internal ablation protocol, while Table 7, Table 8 and Table 9 provide the detailed WaterScenes ablation. Nevertheless, these analyses do not establish unrestricted domain generalization; the result is best interpreted as cross-dataset and coarse condition-level consistency within the evaluated RGB water-scene settings.

**Role of Qualitative Evidence.** Near-identical boxes for jointly detected objects do not imply the absence of a meaningful qualitative advantage. Correct detectors should converge on similar geometry for the same visible object; the intended advantage is not displacement of shared boxes but recovery of difficult objects omitted by competing models. At the identical 0.3 threshold, the additional WaterMamba detections show this operating-point behavior. A fixed threshold necessarily converts continuous confidence scores into retained and discarded predictions, so the fact that a visible difference crosses the threshold is not, by itself, an alternative explanation that invalidates the detection. More importantly, the advantage persists in confidence-ranked AP over the complete evaluation sets and is especially pronounced in AP_*S*_, which cannot be produced by the display threshold. We therefore treat the figures as supportive qualitative evidence that is consistent with, but not a substitute for, the full-set quantitative results. We make no separate claim of superior confidence calibration from these panels.

**Robustness and Failure Cases.** The fixed-threshold panels reveal that extremely small targets may still be missed when their boundaries are nearly indistinguishable from wave texture, and strong specular highlights can produce ambiguous predictions. Although Table 4 provides a coarse condition-level comparison, the current experiments do not report formal calibration error, sequence-level robustness, or exhaustive weather-stratified validation. We therefore do not claim improved calibration or universal robustness to night, turbidity, fog, or glare. Additional temporal cues, calibration analysis, and sequence-independent weather-stratified validation are required before making those claims.

**Limitations and Future Work.** WaterScenes and IWHR use image-level splits, and sequence-level independence has not been established for the offline LaRS split. Similarity between training and evaluation samples therefore cannot be fully ruled out. WaterScenes validation data were also used for hyperparameter selection and final reporting, so model-selection bias remains possible. The tables report single-run results without mean ± standard deviation across random seeds; the smaller module-level gains have not been shown to exceed training variation.

The condition-level analysis relies on available metadata or manual tags and does not constitute a controlled, weather-balanced evaluation. The fixed-threshold panels illustrate detection behavior but do not measure confidence calibration. FDA introduces FFT/IFFT overhead and may attenuate useful texture, while the present model does not address temporal consistency. The reported 78.4 FPS was measured on an NVIDIA A100; latency, peak memory use, and power consumption on ASV-oriented embedded hardware remain unevaluated. Future work should prioritize sequence-independent testing, repeated-seed evaluation, condition-stratified analysis, and embedded-platform measurements.

## 6. Conclusions

We have presented WaterMamba, a hybrid Mamba-based detection transformer for small-target river obstacle detection. The method combines MRFFI-based state-space feature extraction, learned spatial–spectral encoding through HybridMamba and FDA, TCWB multi-scale fusion, and ASDL scale-aware optimization.

Under the unified RGB-only protocol, WaterMamba improves AP_*S*_ over RT-DETR-L by 4.5 points on WaterScenes and 4.8 points on both LaRS and IWHR. The WaterScenes ablations provide the component-level evidence: progressive architectural replacement, deletion-based replacement, and HybridMamba submodule analysis all show that the final model benefits from efficient state-space feature extraction, spatial-spectral encoding, triple-channel cross-scale fusion, and scale-aware supervision. WaterMamba uses 28.6 M parameters, requires 96.2 G FLOPs, and reaches 78.4 FPS on an NVIDIA A100. This server-GPU throughput does not establish embedded real-time performance.

Evaluation is limited to three RGB benchmarks and server-GPU timing, without sequence-independent validation, formal calibration analysis, temporal modeling, repeated-run uncertainty, or embedded-platform latency. The fixed-threshold examples illustrate detection behavior and failure cases, while full-set metrics and ablations support the quantitative comparisons. Under the evaluated conditions, the results support learned spectral modulation as a complement to spatial state-space modeling for water-scene obstacle detection.

## Figures and Tables

**Table 1:** Summary of the object-detection protocols used in this study.

Dataset	Images Used	Train/Test	Detection Labels	Annotation Source
WaterScenes	54,000	43,200/10,800	8 working categories	Pre-converted bbox annotations
LaRS	4000	3200/800	Instance obstacles	Boxes from available panoptic masks
IWHR	3000	2400/600	Floater	Public bbox annotations

**Table 7:** Progressive performance ablation on the WaterScenes dataset. Each architectural entry replaces the corresponding operation in the preceding configuration; ASDL is applied only during training.

Configuration	mAP_50_	mAP_50:95_	AP_*S*_	Params (M)	FLOPs (G)
RT-DETR-L (Baseline)	74.5	45.9	32.3	32.0	108.0
MobileMamba backbone w/MRFFI	75.6	46.7	33.8	29.4	98.6
HybridMamba encoder	76.3	47.4	35.0	29.0	97.4
TCWB cross-scale fusion	76.8	47.9	35.9	28.6	96.2
ASDL during training (Full Model)	**77.3**	**48.6**	**36.8**	28.6	96.2

Note: Bold indicates the best performance value in each metric column.

**Table 8:** Deletion-based performance ablation from the full WaterMamba configuration on WaterScenes. The restored baseline operations for the MobileMamba, HybridMamba, and TCWB variants make this a replacement-based analysis.

Configuration	mAP_50_	mAP_50:95_	AP_*S*_	Params (M)	FLOPs (G)
Full WaterMamba	**77.3**	**48.6**	**36.8**	28.6	96.2
w/o MobileMamba + MRFFI	76.1	47.5	34.7	31.8	105.4
w/o HybridMamba	76.6	47.8	35.4	28.9	97.3
w/o FDA branch	76.6	47.8	35.7	28.5	95.7
w/o TCWB	76.7	47.8	35.8	28.8	96.8
w/o ASDL	76.8	47.9	35.9	28.6	96.2

Note: Bold indicates the best performance value in each metric column.

## Data Availability

The WaterScenes dataset is publicly available at https://github.com/WaterScenes/WaterScenes (accessed on 22 September 2026). The LaRS dataset is available at https://lojzezust.github.io/lars-dataset/ (accessed on 22 September 2026). The IWHR dataset is available at https://doi.org/10.6084/m9.figshare.27376851.v1 (accessed on 22 September 2026). The public WaterMamba source package, including model code, dataset readers, training/evaluation/inference entry points, and configurations for the three datasets, is available at https://github.com/Lixiang733/WaterMamba (accessed on 22 September 2026). The package has been revised to include a COCO-style evaluator and explicit notes on training settings and converted-label assumptions. Public repository timestamps document dissemination and package maintenance; they do not identify the execution time of private/local experiments.

## Abbreviations

The following abbreviations are used in this manuscript:

SSM	State Space Model
VSSM	Visual State Space Model
MRFFI	Multi-Receptive Field Feature Interaction
MRFB	Multi-Range Fusion Block
MBHA	Multi-Branch Hybrid Attention
FDA	Frequency-Domain Attention
TCWB	Triple-Channel Window Block
TCWA	Triple-Channel Window Attention
ASDL	Adaptive Scale-Aware Detection Loss
DETR	Detection Transformer
RT-DETR	Real-Time Detection Transformer
FPN	Feature Pyramid Network
mAP	Mean Average Precision
AP_*S*_	Average Precision for Small Objects
CIoU	Complete Intersection over Union
FFT	Fast Fourier Transform
